# A Novel Plant-Derived Biopesticide Mitigates *Fusarium* Root Rot of *Angelica sinensis* by Modulating the Rhizosphere Microbiome and Root Metabolome

**DOI:** 10.3390/plants13162180

**Published:** 2024-08-06

**Authors:** Qi Liu, Waqar Ahmed, Guoli Li, Yilin He, Mohamed Mohany, Zhaoyu Li, Tong Shen

**Affiliations:** 1 Research Institute, Lanzhou Jiaotong University, Lanzhou 730070, China; 2 Guangdong Province Key Laboratory of Microbial Signals and Disease Control, College of Plant Protection, South China Agricultural University, Guangzhou 510642, China; 3 Department of Pharmacology and Toxicology, College of Pharmacy, King Saud University, Riyadh 11451, Saudi Arabia

**Keywords:** *Angelica sinensis*, disease suppression, biological control, rhizosphere microbiome modulation, root metabolomics

## Abstract

*Fusarium* root rot caused by the *Fusarium* species complex significantly affects the yield and quality of *Angelica sinensis*, a valuable medicinal herb. Traditional management primarily relies on chemical fungicides, which have led to pathogen resistance, environmental hazards, and concerns regarding public health and the active components in *A. sinensis*. This study explores the efficacy of a novel plant-derived biopesticide Shi Chuang Zhi Feng Ning (T1; SCZFN), alongside *Bacillus subtilis* wettable powder (T2) and a chemical fungicide (T3), in controlling root rot and understanding their impacts on the rhizosphere microbial community and root metabolome. Results of the field experiment demonstrated that treatments T1 and T3 achieved control efficiencies of 73.17% and 75.45%, respectively, significantly outperforming T2 (39.99%) and the control. High-throughput sequencing revealed that all treatments altered the diversity and structure of microbial communities, with T1 and T2 reducing the abundance of taxa linked to root rot, such as *Muribaculaceae* spp., *Humicola* spp., *Fusarium* spp., and *Mycochlamys* spp. Treatment T1 notably enhanced beneficial bacterial taxa, including *Acidobacteria* spp., *Nitrospira* spp., and *Pedosphaeraceae* spp., involved in carbon cycling and plant growth promotion. Metabolomic analysis identified 39, 105, and 45 differentially expressed metabolites (DEMs) across the treatments, demonstrating T1’s potential to modulate the root metabolome effectively. Further, a correlation analysis demonstrated a stronger correlation between distinct microorganisms with significant influence and DEMs of T1 treatment compared to other treatments. These findings underscore biopesticide SCZFN’s role in enhancing plant health and disease suppression in *A. sinensis*, providing insights into its biocontrol mechanisms and supporting the development of sustainable disease management strategies in its cultivation.

## 1. Introduction

*Angelica sinensis* (Oliv.) Diels, a member of the Umbelliferae family, is commonly known as Dang Gui in China. It is a well-known traditional Chinese medicinal herb with a long history of cultivation and is famous for its medicinal value [1]. *A. sinensis* is widely used in the medical field and contains over 80 essential chemical components such as volatile oils (e.g., ligustilide and n-butenolactone), organic acids (e.g., vanillic acid and ferulic acid), flavonoids, terpenoids, polysaccharides, and others [2]. It exhibits superior biological activities, including anti-tumor, anti-inflammatory, and antioxidant properties, as well as uterine disorders and blood-nourishing effects [3], and ranks among China’s top 5 herbal medicines [4]. In addition to its medicinal properties, *A. sinensis* has been exported to more than 20 countries and regions, including America, Europe, Taiwan, Hongkong, and Southeast Asia, as a dietary supplement [5].

Gansu Province in China, mainly Minxian County, is famous for *A. sinensis* production with a long cultivation history of more than 1700 years. With the increase in domestic and international market demands, the cultivation area of *A. sinensis* steadily increases each year. In 2018, the planting area of *A. sinensis* in Gansu Province reached approximately 44,000 hectares, yielding an annual production of about 150,000 tons worth CNY 2.2 billion [6]. Recently, with the expansion of the *A. sinensis* cultivation area and continuous cropping to meet the market demand, root rot disease has become a significant obstacle to healthy crop production and decline in yield and quality [4,5,7]. During the early stage, *A. sinensis* root rot is characterized by the yellowing and wilting of leaves followed by necrosis of the root tissue, which hinders nutrient supply to the aerial parts and ultimately leads to plant mortality due to inadequate nutrition and water availability [4].

Many previous studies reported that multiple genera or species of pathogens can contribute to the occurrence of root rot disease [8,9]. *Fusarium* species are the main culprit and responsible for the occurrence of root rot diseases in many crops [5]. Various *Fusarium* spp. have been reported as the primary pathogen of root rot disease, including *Fusarium tricinctum*, *F. solani, F. oxysporum*, *F. acuminatum,* and *F. avenaceum* [4,5,7]. Similarly, *F. solani*, *F. oxysporum*, and *F. acuminatum* were identified as the primary culprits causing root rot in *Atractylodes macrocephala* and *Astragalus membranaceus* [9]. Many previous studies have reported that root rot is usually a mixed infection of different pathogens, in addition to the primary pathogens, also accompanied by various “helper” auxiliary pathogens, including *Clonostachys rosea* [10], *Cylindrocarpon destructans* [11], and *Alternaria* spp. [8].

There has been limited research on *A. sinensis* root rot, and most of the studies primarily focus on isolating and collecting pathogens, with few studies conducted on effective prevention and control methods. In agricultural production, chemical pesticides such as carbendazim [12], fludioxonil [13], and hymexazol [14] are commonly employed for root rot prevention and control. However, their increased usage in quantity and frequency has resulted in environmental hazards, toxicity to no-targeted organisms, disturbed soil microbial diversity, and developed resistance in pathogens to fungicides [4,15] and increased the incidence of *A. sinensis* root rot [5]. So, there is an urgent need to delve into eco-friendly control measures in the form of biological control using beneficial microbes or their active bioproducts in sustainable agriculture [16,17].

Biocontrol agents, especially bacteria and fungi, significantly mitigate the incidence of root rot disease and are commercially available in different countries [4,7]. For example, members of bacterial genera from *Bacillus* spp., *Pseudomonas* spp., and *Streptomyces* spp. improve plant growth and significantly control root rot disease in strawberries and beans [18,19]. Similarly, it has been reported that *B. tequilensis* SY89 and *Paenibacillus polymyxa* YF could secrete antimicrobial substances (polypeptides, lipopeptides, and polyketides) and significantly reduced *A. sinensis* root rot by 61.54% and 65.38%, respectively [4]. Besides the application of a single-strain biocontrol agent, the microbial consortium successfully reduces the root rot incidence by reshaping the rhizosphere microbiome and decreasing the pathogen load [20,21]. Many previous studies have reported that the occurrence of root rot disease is directly correlated with changes in the rhizosphere microbial community and alterations in the abundance of key pathogens [20,22,23]. Thus, analyzing the composition and distribution of rhizosphere microbial communities is crucial for their effective management to mitigate soilborne plant diseases, enhance plant growth performance, and optimize agricultural yields [24,25,26].

In general, disease control primarily involves reducing the relative abundance of pathogens within the soil microbial community, thereby allowing other microorganisms to occupy the original ecological niche of the pathogens [20,27]. Additionally, disease control can be achieved by increasing the prevalence of beneficial microbial flora capable of resisting or inhibiting soilborne pathogens [25,28]. The interaction between the root system and rhizosphere microorganisms plays a pivotal role in promoting the growth and enhancing the quality formation of medicinal plants [29]. Plant secondary metabolites, particularly root exudates, can significantly influence the dynamics of soil microbial communities [30]. Moreover, these compounds encompass bioactive constituents that can impede pathogen growth or recruit beneficial microorganisms for enhancing plant growth and inducing systemic defense mechanisms against pathogens [31,32]. Additionally, plant secondary metabolites are subject to modulation by both biotic factors (microbial communities and population competition) [33] and abiotic factors (soil properties and climate conditions) [34]. Thus, it is suggested that root rot has become a significant obstacle to the cultivation of *A. sinensis*, which seriously threatens its production.

Therefore, it is crucial to develop a biological pesticide that can effectively manage the occurrence of *Angelica* root rot through biological control methods. The biopesticide Shi Chuang Zhi Feng Ning (SCZFN; drug registration number: PD20140941) is extracted from a traditional Chinese medicinal plant with an active ingredient of 5% carvacrol incorporating various antibacterial and insecticidal activities. The biopesticide SCZFN has been applied to over 200,000 hectares of Chinese medicinal materials, vegetables, potatoes, tea, and other crops. It exhibits remarkable efficacy in controlling various plant diseases, particularly *A. sinensis* root rot prevention and control. However, the mechanism underlying the control of root rot in the field by SCZFN remains inadequately elucidated. In this study, we investigated the changes in the rhizosphere soil microbial community and secondary metabolites of *A. sinensis* roots to elucidate the preventive and therapeutic mechanisms of SCZFN against *A. sinensis* root rot, along with *Bacillus subtilis* wettable powder as a microbial bioagent and a commonly used chemical pesticide among farmers. We assumed that the investigation of rhizosphere microbial communities could unveil the interaction between SCZFN and pathogens, as well as the correlation between pathogens and other microorganisms. Furthermore, exploring the secondary metabolites of the *A. sinensis* root system can elucidate the impact of SCZFN on the diversity and metabolic pathways of these secondary metabolites.

## 2. Results

### 2.1. Assessment of Different Treatments on Biocontrol Efficiency of Angelica sinensis Root Rot

To systematically evaluate the efficacy of different treatments in controlling *Angelica sinensis* root rot, we assessed the disease index (DI), disease incidence (Di), and control effect (CE) at the end of the experiment under different treatments (Table 1). Results demonstrated that treatments T1 and T3 had the lowest values of DI (19.67% and 18.00%) and Di (28.33% and 23.61%), as compared to T2 (DI; 44.00% and Di; 47.83%) and CK (DI; 73.33% and Di; 92.00%). Treatment T1 showed the best CE of 73.17%, which is similar to the CE of treatment T3 (75.45%) and significantly higher than the CE of T2 (39.99%).

### 2.2. Effect of Different Treatments on the Angelica sinensis Rhizosphere Microbiome Assembly

**Figure 1:**
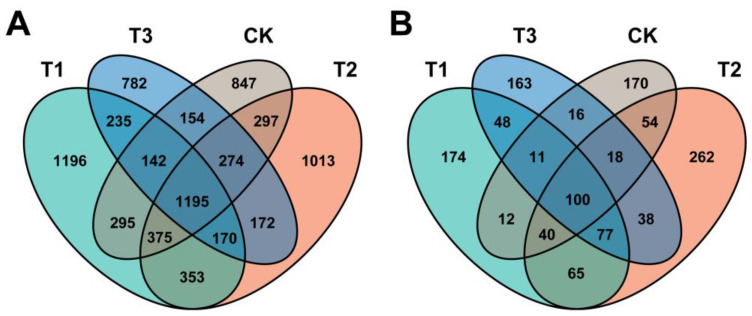
**Venn diagram of the various treatments displays the distribution of common and shared amplicon sequence variants (ASVs).** The presence of intersecting numbers signifies shared ASVs, while the absence of intersections indicates unique ASVs. The ASVs distribution in bacteria (**A**), and the ASVs distribution in fungi (**B**). Application of biopesticide shi chuang zhi feng ning (SCZFN) (T1), application of *Bacillus subtilis* wettable powder (T2), application of fungicide Apron Advance (T3), application of water (CK).

A total of 12 rhizosphere soil samples were subjected to an Illumina NovaSeq platform to generate 16S and ITS paired-end sequencing. The data collected from Illumina sequencing were processed for quality control, barcode and primer sequences removal, and chimeras removal to obtain clean data. The data related to raw reads, effective tags, Q20 and 30, and effective tag (%) of 16S and ITS gene sequencing are shown in Tables S1 and S2. Amplicon sequence variants (ASVs) obtained through DADA2 analysis clustered clean data at a 100% sequence similarity level for taxonomic annotation. The ASV results of bacteria and fungi after denoise were classified into common and unique ASVs according to different treatments, as shown in the Venn diagram (Figure 1). Analysis of ASVs showed a total of 7500 bacterial ASVs, including 3961 in T1, 3849 in T2, 3124 in T3, and 3579 in CK, were found under different treatments. Among them, 1196, 1013, 782, and 847 ASVs were unique in T1, T2, T3, and CK, respectively, and 1195 ASVs were common in all treatments (Figure 1A). A total of 1248 fungal ASVs were recovered, of which 527, 654, 417, and 174 ASVs belonged to T1, T2, T3, and CK, respectively. Furthermore, ASV analysis showed that 174, 262, 163, and 170 were recovered as unique ASVs in T1, T2, T3, and CK, respectively, with 100 ASVs as common among all treatments (Figure 1B). Then, we annotated the species classification of bacteria and fungi based on all ASVs and investigated the phylogenetic relationships of the top 100 representative sequences at the genus level through multiple sequence alignment, respectively (Figure S1A,B).

### 2.3. Analysis of Distribution and Abundance of Rhizosphere Microbial Communities at the Phyla Level

**Figure 2:**
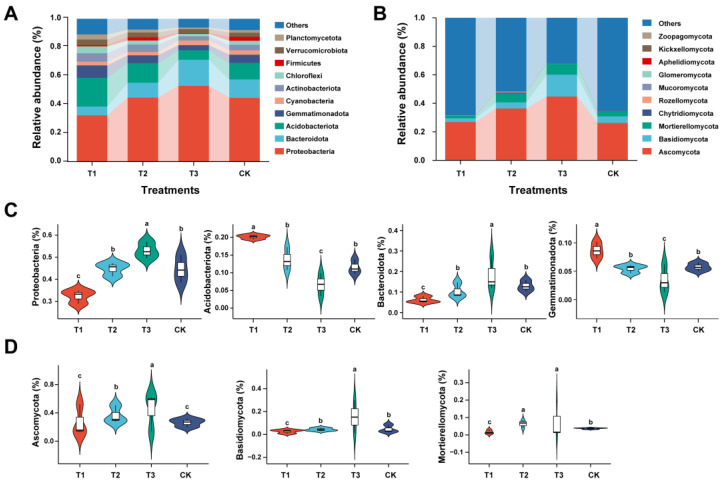
**The dynamics of most dominant bacterial and fungal communities at the phyla level.** Relative abundance bar plots for the top 10 most abundant bacterial (**A**) and fungal (**B**) phyla. The box plot shows the significant difference and relative abundance of the differentially abundant bacterial (**C**) and fungal (**D**) phyla under different treatments. The lowercase letters on each box plot display significant differences among treatments (Wilcoxon test, *p* < 0.05). Application of SCZFN (T1), application of *Bacillus subtilis* wettable powder (T2), application of fungicide Apron Advance (T3), and application of water (CK).

To better understand the impact of the three treatments on the microbial community structure and composition, we conducted a statistical analysis for the top ten most abundant bacterial and fungal phyla (Figure 2). The bar plot histograms were generated to reveal the classifications of abundant ASVs of bacteria (Figure 2A) and fungi (Figure 2B) at the phylum level. Bacterial phyla such as Proteobacteria, Acidobacteriota, Bacteroidota, and Gemmatimonadota dominated the soil bacterial communities with a relative abundance (RA) of 67.4%, 74.4%, 81.5%, and 74.8% in T1, T2, T3, and CK, respectively (Table S3). Proteobacteria and Bacteroidota were found in low RA in the rhizosphere soil of T1 compared with T2, T3, and CK. In contrast, there were several phyla, such as Acidobacteriota and Gemmatimonadota, that were present in high RAs in the rhizosphere soil of T1 than T2, T3, and CK (Figure 2C). Fungal phyla such as Ascomycota, Basidiomycota, and Mortierellomycota dominated the soil fungal communities, and their RA accounted for more than 99% in T1, T3, and CK except for T2 (98.76%) (Table S4). Fungal phyla such as Basidiomycota and Mortierellomycota exhibited lower relative abundances in T1 compared to other treatments and CK. Further, T1 demonstrated a significantly reduced RA of Ascomycota compared to T2 and T3 (Figure 2D).

### 2.4. Relative Abundance Analysis of Rhizosphere Microbial Community Composition at the Genus Level

**Figure 3:**
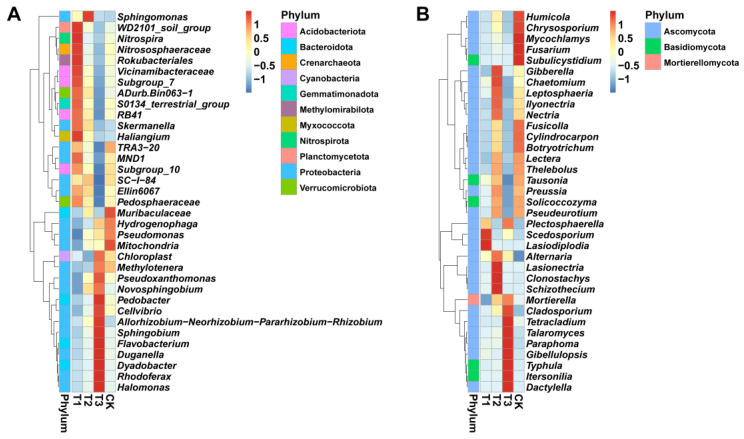
**The analysis of the patterns of taxonomic distribution and soil microbial composition at the genus level.** The relative abundance heat maps and phylum-level cluster maps of the top 35 bacteria (**A**) and fungi (**B**) under different treatments. Application of SCZFN (T1), application of *Bacillus subtilis* wettable powder (T2), application of fungicide Apron Advance (T3), and application of water (CK).

We conducted a genera-level analysis to better clarify the patterns of taxonomic distribution and composition in the rhizosphere of *Angelica sinensis* under different treatments (T1, T2, T3, and CK). Based on the species information and RA, heatmaps were generated to display the RA of the top 35 bacterial and fungal genera under different treatments (Figure 3). Further analysis of bacterial communities with different RAs revealed that the rhizosphere of treatment T1 was significantly enriched with *Nitrosphera* spp., *Nitrososphaeraceae* spp., *Pedosphaeraceae* spp., *Rokubacteriales* spp., *Vicinamibacteraceae* spp., *RB41* spp., *Haliangium* spp., *Subgroup_7* spp., and *Subgroup_10* spp. as compared to T2, T3, and CK (Figure 3A). *Haliangium* spp. is the main predatory bacteria, and interestingly, the RA of *Haliangium* spp. was significantly increased under the T1 treatment, which was not observed in other treatments. Moreover, T1 treatment significantly increased the *Pedosphaeraceae* spp. and *ADurb.Bin063–1* spp. belonging to the *Verrucomicrobiota*, and *Pedosphaeraceae* spp. plays a key role in the biogeochemical cycles of organic materials. Similarly, the RA of *Nitrospira*, an important genus of bacteria revealed to simplify nitrogen nitrification, increased in T1 treatment, which was also different from other treatments.

On the other hand, the result of fungal communities showed that T1, T2, and T3 treatments could significantly reduce the important pathogenic fungal genus of *Fusarium* that causes root rot disease and the genus of fungi with high degradation efficiency effects on humus, lignin, and cellulose, including *Humicola* spp. and *Chrysosporium* spp. (Figure 3B). Typically, these genera are widely considered dominant fungi that cause continuous cropping issues and root rot disease. In addition, it can be seen from Figure 3B that fungal genera present in high RA in CK were significantly reduced in T1 and T3 treatments. These results agree with the experimental results obtained in the field that T1 and T3 treatments significantly inhibited the occurrence of *A. sinensis* root rot disease.

### 2.5. Assessment of Differences in Rhizosphere Microbial Community Diversity and Structure

**Figure 4:**
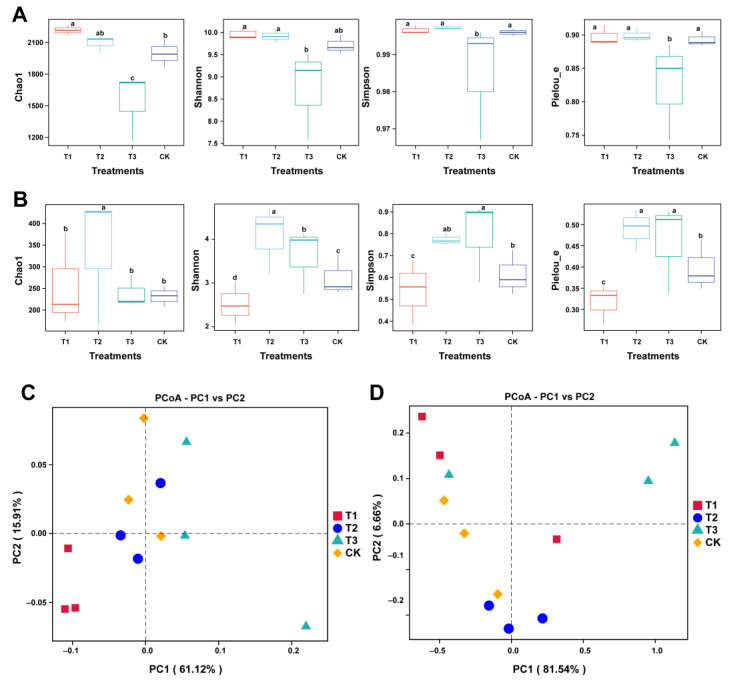
**Assessment of microbial community diversity and structure difference under different treatments.** Box plot showing the alpha diversity indices of bacterial (**A**) and fungal (**B**) communities under different treatments. Alpha diversity indices include Chao 1, Shannon, Simpson, and Pielou evenness. Different lowercase letters on each box plot represent the significant differences among treatments according to the Wilcoxon test at *p* < 0.05. Principal coordinate analysis (PCoA) based on the Bray–Curtis distance matrix demonstrates the separation between soil bacterial and fungal communities under different treatments. PCoA for bacterial (**C**) and fungal (**D**) communities. Application of SCZFN (T1), application of *Bacillus subtilis* wettable powder (T2), application of fungicide Apron Advance (T3), and application of water (CK).

We analyzed the diversity and structure of the rhizosphere microbial community of *A. sinensis* to better explore the changes in community composition under different treatments (Figure 4). The alpha diversity indices (within-sample diversity) were calculated to quantify the species richness of rhizosphere microbial communities under different treatments (Figure 4A,B). The result showed that the Chao 1, Shannon, Simpson, and Pileou indices of bacterial communities were found to be significantly higher in T1, T2, and CK as compared to T3, and no significant difference was observed between T1, T2, and CK, except for Chao 1 index (Figure 4A). However, the alpha diversity indices of fungal communities showed an opposite trend compared to bacterial communities. The values of Chao 1, Shannon, Simpson, and Pileou indices were significantly decreased in T1 than that of T2, T3, and CK (Figure 4B). Further, Principal coordinate analysis (PCoA), based on the Bray-Curtis dissimilarity matrix and ASV relative abundance distribution, was used to assess the changes in the structure of bacterial and fungal communities under different treatments. According to the PCoA results, the first two showed a total of 77.03% and 88.2% variations in the structure of rhizosphere bacterial (PERMANOVA, R^2^; 0.638341 and *p* = 0.001) and fungal (PERMANOVA, R^2^; 0.56681 and *p* = 0.004) communities, respectively, under different treatments (Figure 4C,D).

### 2.6. Characteristics of Angelica sinensis Intra-Kingdom Rhizosphere Microbial Co-Occurrence Network

**Figure 5:**
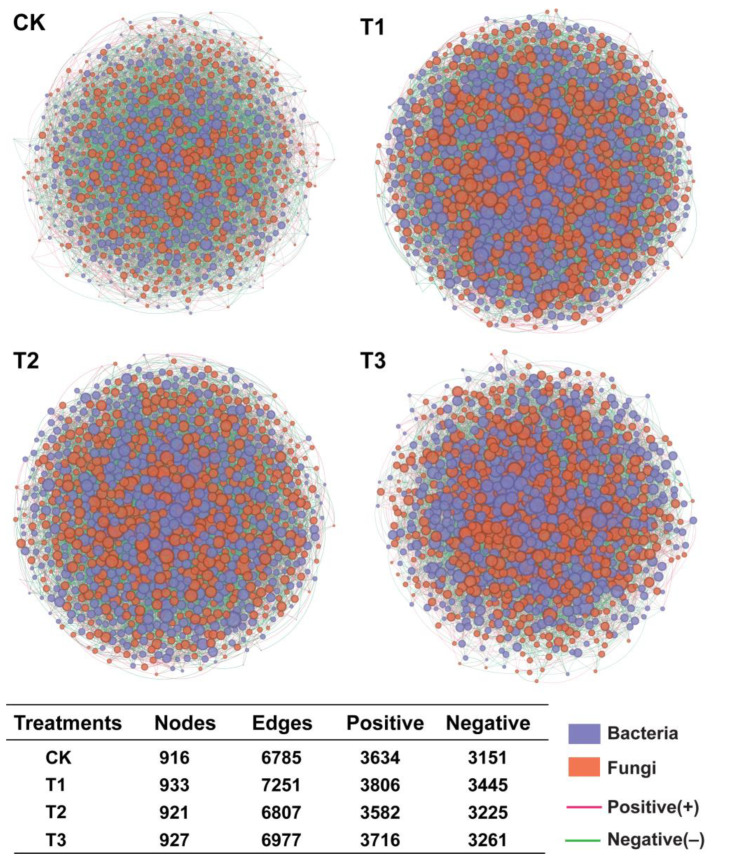
**Co-occurrence networks analysis of bacterial and fungal communities at genus level under different treatments.** Nodes represent microbial genera, and edges represent the interaction between microbes within a specific treatment, including the number of positive and negative edges. Application of SCZFN (T1), application of *Bacillus subtilis* wettable powder (T2), application of fungicide Apron Advance (T3), and application of water (CK).

The interkingdom co-occurrence network analysis was performed to investigate the impact of different treatments on bacterial-fungal interactions (Figure 5). The co-occurrence network showed that the number of nodes was similar under all treatments without significant differences (CK = 916, T1 = 933, T2 = 921, and T3 = 927). The number of edges (total; 7251, positive; 3806, and negative; 3445) of T1 was significantly higher than other treatments, including T2 (total; 6807, positive; 3582, and negative; 3225), T3 (total; 6977, positive; 3716, and negative; 3261), and CK (total; 6785, positive; 3634, and negative; 3151). These results suggested that the abundance of different microorganisms under treatments T1, T2, and T3 was increased compared with CK (the circle size represents the absolute abundance of different bacterial and fungal genera). Specifically, comparing the co-occurrence network among treatments showed that T1 had higher network connectivity and complex microbial interaction than other treatments. This indicated that T1 inhibited pathogenic bacteria from occupying the ecological niche in the rhizosphere of *A. sinensis*, resulting in a more complex microbial interkingdom network than CK.

### 2.7. Correlation Analysis of Microbial Communities and Disease Incidence

**Figure 6:**
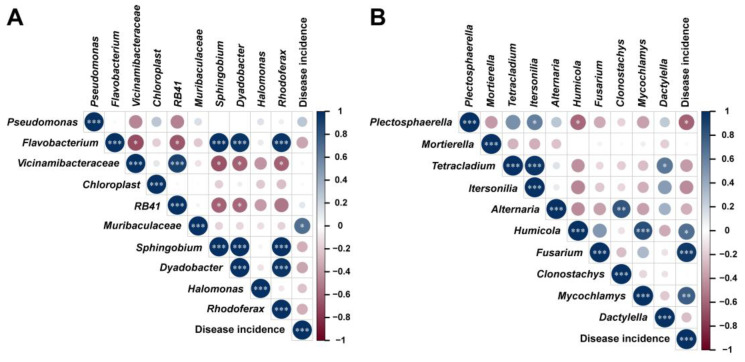
**Correlation analysis between Top 10 bacterial-fungal genera and disease incidence according to Pearson correlation coefficient (PCC, *p* < 0.05).** PCC between bacterial genera and disease incidence (**A**), and PCC between fungal genera and disease incidence (**B**). Asterisks indicates significant differences (* *p* < 0.05; ** *p* < 0.01; *** *p* < 0.001).

To further explore the relationship between disease occurrence and results of microbial interkingdom co-occurrence network, a correlation analysis was performed at the genera level according to the Pearson correlation coefficient (PCC; Figure 6). The PCC results revealed that bacterial genera *Muribaculaceae* spp. (Figure 6A) and fungal genera *Humicola* spp., *Fusarium* spp., and *Mycochlamys* spp. (Figure 6B) were positively correlated (*p* < 0.05) with the disease incidence. The correlation results were consistent with those in Figure 3A,B, as these microorganisms were significantly enriched in CK compared with other treatments. These results showed that the primary pathogen causing root rot in *A. sinensis* is the genus *Fusarium*, and combined results of correlation analysis suggested that *Muribaculaceae* (coefficient = 0.6964; *p*-value *=* 0.012) spp., *Humicola* (coefficient = 0.6892; *p*-value = 0.013) spp., and *Mycochlamys* (coefficient = 0.7689; *p*-value *=* 0.0034) spp., play a role of assisting microorganisms, which provides a conducive environment for *Fusarium* spp., to infect the roots of *A. sinensis*.

### 2.8. Integrated Characteristics of Metabolomics of Angelica sinensis

We identified the metabolites of all treatments and retained the results of a 30% lower coefficient of variance through quality control (QC) (Figure S2). Finally, a total of 799 metabolites were counted, including 472 positive ion modes (POS) and 327 negative ion modes (NEG). The metabolites were chemically classified by comparison with three databases (mzCloud, mzVault, and MassList). The metabolites were classified into 10 categories in POS (Figure S3A) and 7 categories in NEG (Figure S3B). In two ion modes, lipids and lipid-like molecules, as well as organic acids and derivatives, were the most diverse metabolites and accounted for more than 50% of the total categories. The principal component analysis (PCA) was used for all mass spectrum peaks of treatments and QC samples. The results showed that the first two axes explained, in total, 31.19% of POS (Figure S4A) and 36.08% of NEG (Figure S4B) variations in the metabolites, respectively. In addition, QC samples of both POS and NEG are gathered, and the aggregation locations of the different treatment groups are a certain distance from CK. This suggested that treatments and QC are of high quality, and the metabolite types and their accumulation in *A. sinensis* were significantly affected by different treatments. The results of PLS-DA are shown in Figure S5.

Meanwhile, the functional and classification annotations of all identified metabolites were made by comparison with three databases (KEGG (KEGG PATHWAY), HMDB (Human Metabolome Database) and LMPD (LIPID MAPS Structure Database)) to clarify the functional properties and types of these metabolites. The comparison results based on the KEGG database showed that all metabolites in POS were clustered in 4 categories: cellular processes, environmental information processing, genetic information processing, and metabolism (Figure S6A). Among them, the metabolites of metabolism occupy the highest proportion in all categories; the metabolites in secondary class global and overview maps were significantly higher than those compared with others. Conversely, metabolites in NEG were clustered in three categories, including environmental information processing, genetic information processing, and metabolism (Figure S6B). Similar to POS, the metabolite of Metabolism in NEG occupies the highest proportion in all categories, and global and overview maps were significantly higher than others in the secondary class.

Interestingly, the classification and function of the results compared with the HMDB database were consistent with the above results shown in Figure S3. The order of metabolite numbers in POS was slightly different (Figure S6C), but the order of metabolite numbers in NEG was consistent with Figure S3B (Figure S6D). Finally, compared with the LMPD database, both POS and NEG were clustered in the five primary categories: fatty acyls, glycerophospholipids, polyketides, prenol lipids, and sterol lipids. Among them, the number of metabolites in fatty acyls was the highest in POS (Figure S6E), and the number of metabolites in the category glycerophospholipids was the highest in NEG (Figure S6F).

### 2.9. Assessment of Variations in Angelica sinensis Metabolites under Different Treatments

**Figure 7:**
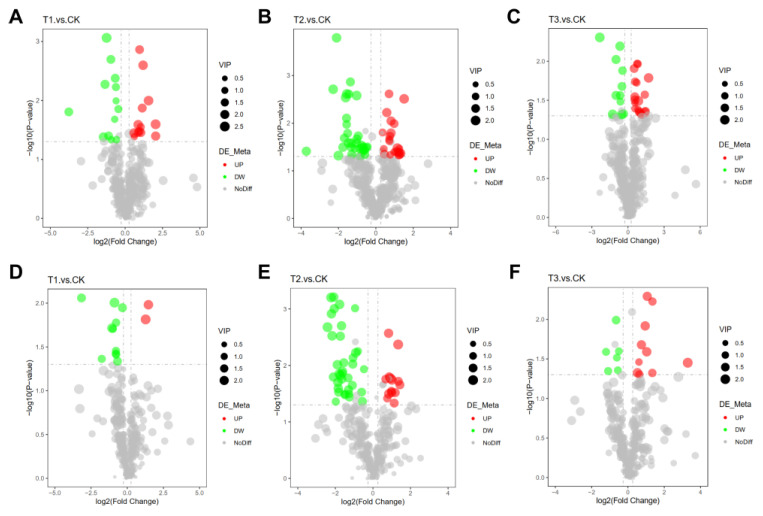
**The analysis of d differentially expressed metabolites after comparison of different treatments and controls.** The presence of intersecting numbers signifies shared DEMs, while the absence of intersections indicates unique DEMs. Volcano map of the overall distribution of DEMs. (**A**–**C**) DEMs of different treatments compared to CK in positive mode, (**D**–**F**) DEMs of different treatments compared to CK in negative mode. The horizontal coordinate represents the difference in multiple changes of metabolites in different groups (log2(fold change)), and the vertical coordinate represents the difference in significance level (−log10(*p*-value)). Each point represents a metabolite. Significantly up-regulated metabolites are represented by red dots, and significantly down-regulated metabolites are represented by green dots. The size of the dot represents the VIP value. Application of SCZFN (T1), application of *Bacillus subtilis* wettable powder (T2), application of fungicide Apron Advance (T3), and application of water (CK).

To clarify the quantity and type of significantly different metabolites between treatments and CK, we screened out the differentially expressed metabolites (DEMs) between treatments and controls. The results showed that compared with CK, there were 162 DEMs in all treatments, including 39 DEMs in T1 vs. CK, 45 DEMs in T3 vs. CK, and 105 DEMs in T2 vs. CK was significantly higher than others. Meanwhile, among all DEMs, 27 unique metabolites were detected in T1 vs. CK, 28 unique metabolites in T2 vs. CK, and 81 unique metabolites were significantly higher than others in T2 vs. CK. In POS, 26, 55, and 29 DEMs were present in T1, T2, and T3, respectively, compared to the CK. Among all DEMs, T1 vs. CK showed 13 up-regulation and 13 down-regulation; T2 vs. CK showed 22 up-regulated and 33 downregulated; T3 vs. CK showed 18 up-regulated and 11 downregulated, respectively. In NEG, 13, 50, and 16 DEMs were responded to T1, T2, and T3, respectively, compared to the CK. T1 vs. CK showed 2 up-regulation and 11 down-regulation; T2 vs. CK (14 up-regulated and 36 downregulated), T3 vs. CK (10 up-regulated and 6 down-regulated), respectively (Figure 7, Table S5). Then, we performed hierarchical clustering analysis (HCA) for all the DEMs between the obtained comparisons (Figure S7). In POS, metabolites were divided into four main categories at the first level according to the functions or metabolic processes involved. From top to bottom, the first type of T3 treatment had the most significant difference from CK, and almost all the metabolite levels were up-regulated compared with CK. The remaining categories showed the most significant difference between T2 treatment and CK; all of them showed different up-down levels from the CK. Similarly, in NEG, among the three primary classifications, T2 treatment showed a significant difference in up-down-regulation compared to CK. In the first two categories, from top to bottom, except for the third category, treatment T3 was significantly up-regulated compared to CK. These results indicated that T2 treatment had a much higher effect on the metabolism of *A. sinensis* than other treatments.

### 2.10. KEGG Enrichment Pathways Analysis for DEMs

**Figure 8:**
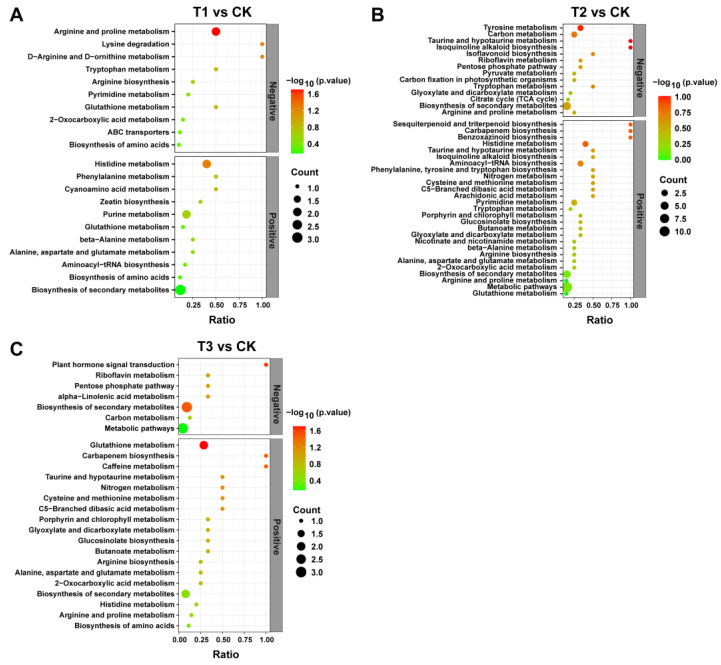
**Analysis of KEGG enrichment pathway between treatments compared to CK in two ion modes (A–C).** The horizontal coordinate is the ratio of the number of differentiated metabolites in the corresponding metabolic pathway to the total number of identified metabolites in the pathway, and the vertical coordinate represents the difference in significance level (−log10(*p*-value)). The size of the dots represents the number of differentiated metabolites in the corresponding pathway. Application of SCZFN (T1), application of *Bacillus subtilis* wettable powder (T2), application of fungicide Apron Advance (T3), and application of water (CK).

To explore the effects of different treatments on the metabolic pathways of *A. sinensis*, we used the KEGG ID for DEMs to derive the metabolic enrichment pathways. The results showed 21 enriched metabolic pathways in T1 compared to CK, including 11 in POS and 10 in NEG (Figure 8A); among them, the significantly enriched pathway was arginine and proline metabolism (*p*-value = 0.01982). In T2 vs. CK, there were 41 enriched metabolic pathways, including 14 in POS and 27 in NEG (Figure 8B). In T3 vs. CK, there were 25 enriched metabolic pathways, including 19 in POS and 7 in NEG (Figure 8C). Among the enriched pathways, the significantly enriched pathway was glutathione metabolism (*p*-value = 0.02733), caffeine metabolism (*p*-value = 0.043), carbapenem biosynthesis (*p*-value = 0.043), plant hormone signal transduction (*p*-value = 0.0322) and biosynthesis of secondary metabolites (*p*-value = 0.042), respectively. Treatment T1 mainly affects the metabolism of amino acids, including the metabolism of arginine, proline, histidine, and so on. Treatment T2 mainly affects the biosynthesis of sesquiterpenoid, triterpenoid, carbapenem, benzoxazinoid, and other substances of *A. sinensis* and the metabolism of tyrosine, carbon, and so on. Treatment T3 mainly affects glutathione metabolism, carbapenem biosynthesis, and plant hormone signal transduction. This suggests that different treatments have different effects on the main metabolic pathways of *A. sinensis*.

### 2.11. Correlations between Differential Microorganisms and DEMs

The decline in the incidence of *A. sinensis* root rot cannot be attributed solely to biopesticide treatment, but secondary metabolites also play a crucial role. Therefore, to elucidate alterations in the relationship between secondary metabolites of *A. sinensis* and the soil microbial community under different treatments, as well as gain further insights into the combined impacts of biopesticide treatments and secondary metabolites on soil microbial community structure, the correlations between the soil microbial communities at the genus level and the DEMs were analyzed by PCC method. Initially, we compared the absolute content of different bacteria (DB) and different fungi (DF) in each treatment’s rhizosphere microbiome with the CK. Subsequently, we identified the top 30 DEMs (top 15 for the POS mode and top 15 for the NEG mode) for subsequent association analysis. To gain deeper insights into the interplay between microorganisms and metabolites, we further selected the top 20 distinct microorganisms (10 fungi and 10 bacteria), along with the top 30 DEMs, for correlation analysis.

**Figure 9:**
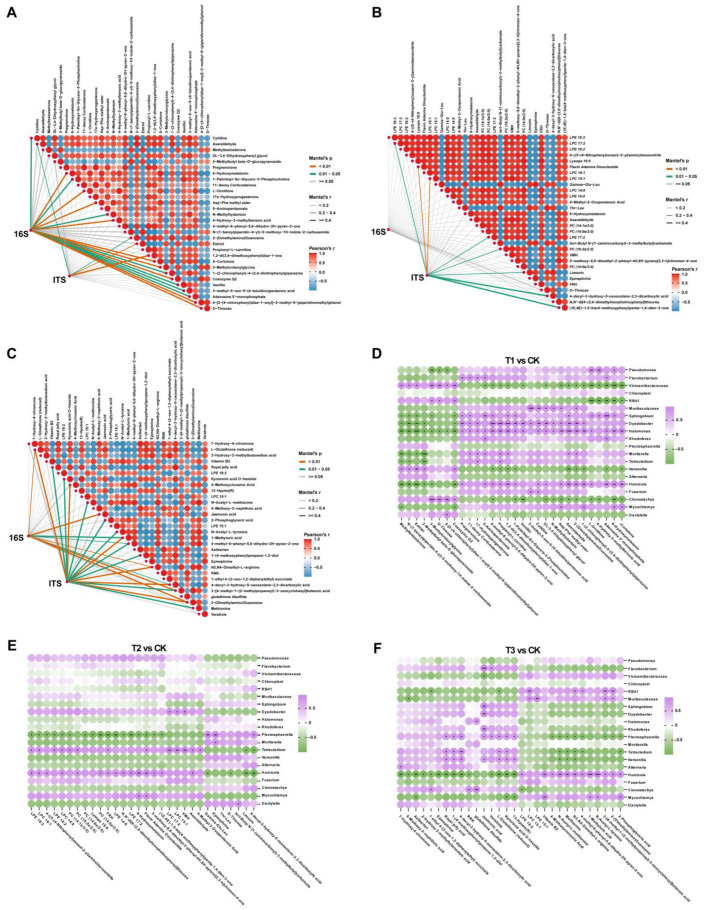
**Interactions between different expressed metabolites and differential microbial communities with different treatments compared to CK.** The interactions among the top 30 distinct metabolites (The top 15 of each of the two ion modes) and diverse microbial communities were compared to CK under various treatments. (**A**–**C**) Correlations of the microbial community and top 30 DEMs were determined using the Mantel test. (**D**–**F**) Correlations analysis of the Top 10 bacterial-fungal genera and Top 30 DEMs according to Pearson correlation coefficient. Asterisks indicates significant differences (* *p* < 0.05; ** *p* < 0.01; *** *p* < 0.001). Application of SCZFN (T1), application of *Bacillus subtilis* wettable powder (T2), application of fungicide Apron Advance (T3), and application of water (CK).

The results demonstrated that the relationship between DB and DEMs exhibited higher complexity and correlation than that between DF and DEMs in T1 vs. CK. Specifically, there was an extremely significant correlation between the variation in DB content and the presence of DEMs 6-hydroxymelatonin, L-ornithine, Asp-Phe methyl ester, 4-hydroxy-3-methylbenzoic acid, β-cortolone, 3-methylcrotonylglycine, coenzyme Q2, 4-[2-(4-chlorophenyl) diaz-1-enyl]-2-methyl-6-(piperidinomethyl) phenol and D-threose, and significantly correlated with metabolites cytidine and 1-palmitoyl-Sn-glycero-3-phosphocholine, etc. On the other hand, fungal communities’ changes were found to be associated with estriol and 1,2-di(3,4-dimethoxy phenyl) diaz-1-ene poles while also exerting an influence on asaraldehyde and 2-(dimethylamino) guanosine (Figure 9A). The further correlation and cluster analysis of the top 20 differential microbial genera and DEMs revealed that bacterial genera including Pseudomonas spp., Flavobacterium spp., *Muribaculaceae* spp., *Sphingobium* spp., *Dyadobacter* spp., *Halomonas* spp. and *Rhodoferax* spp., along with fungi *Mortierella* spp., *Tetracladium* spp., *Humicola* spp., *Fusarium* spp. and *Mycochlamys* spp., exhibited consistent correlations with the DEMs. Specifically, they demonstrated a positive correlation with the right side of pregnenolone and a negative correlation with the left side of coenzyme Q2 among the clustered DEMs. Interestingly, we found that *Sphingobium* spp., *Dyadobacter* spp., and *Halomonas* spp., which are beneficial to the plant and soil environment, as well as *Humicola* spp., which can induce resistance in plants, showed significant correlations with most metabolites (Figure 9D). However, the association analysis results of the microbiome and metabolome in T2 vs. CK revealed non-significant correlations between DEMs and DB. Only 8 DEMs were found to be associated with DF. In comparison, a significant correlation was observed for only two specific DEMs, flavin adenine dinucleotide and LPC 15:1 (Figure 9B). Three DF (*Plectosphaerella* spp., *Tetracladium* spp., and *Humicola* spp.) were significantly associated with most DEMs (Figure 9E). The relationships between DEMs and DF in T3 vs. CK exhibited similarities to those observed in the T1 treatment (Figure 9C). Notably, the top 30 DEMs displayed more intricate associations with differential microorganisms and demonstrated a higher significance level in relation to DEMs than observed in the T2 vs. CK. Among these DEMs, eight were significantly associated with DB, five of which exhibited significant differences. Furthermore, 15 DEMs were significantly associated with DF, 6 showing significant distinctions. In addition, 7 DEMs showed significant associations with both DB and DF (L-glutathione (reduced), 1-methyluric acid, 4-methyl-6-phenyl-5,6-dihydro-2H-pyran-2-one, N3, N4-dimethyl-L-arginine, 4-decyl-3-hydroxy-5-oxooxolane-2,3-dicarboxylic acid, 3-[4-methyl-1-(2-methyl propanol)-3-oxocyclohexyl] butanoic acid and 2-(dimethylamino)guanosine). However, the correlation between the top 20 differential microorganisms and DEMs exhibited significant differences from that of T1 vs. CK (Figure 9F). There was a less pronounced association between T3 and CK in DEMs compared with T1 vs. CK, with only one fungal genus, *Humicola* spp., demonstrating a significant correlation with most differential metabolites.

## 3. Discussion

*Angelica sinensis*, a renowned Chinese herb, holds significant medicinal value in healthcare and medicine [1]. However, the frequent occurrence of root rot and excessive use of chemical pesticides not only diminishes the medicinal properties of *A. sinensis* but also contributes to environmental pollution and potential public health risks [4]. Therefore, it is imperative to urgently identify a biopesticide capable of effectively managing root rot in *A. sinensis* without compromising its valuable medicinal constituents. Since plant-derived biopesticides are sourced from the natural environment, they offer a safer alternative for controlling root rot in *A. sinensis* [35]. The biopesticide Shi Chuang Zhi Feng Ning (SCZFN) created in our laboratory is a plant-based product derived from multiple traditional Chinese medicinal plants. Extensive field tests have demonstrated its exceptional efficacy in controlling root rot and other significant diseases of *A. sinensis* (such as brown spot and stem rot). Field experiment results revealed that the control efficiency of SCZFN against root rot disease is 73.17%, equivalent to the control efficiency (75.45%) of conventional fungicide thiabendazole·fludioxonil·metalaxyl-M 18% FSC. Many previous studies have demonstrated that compared to conventional chemical pesticides, broad-spectrum biological control agents can target multiple pathogens affecting the same host plant during screening, thereby reducing the likelihood of pathogen resistance [4,17]. Although SCZFN demonstrates high efficacy in controlling *A. sinensis* root rot, its underlying disease prevention mechanism remains unreported. Therefore, in this study, we aimed to explore the impact of SCZFN biopesticides on rhizosphere microbial communities and metabolites of *A. sinensis.* We assumed that studying the rhizosphere microbiome and metabolites would provide us with new insights into the biocontrol mechanism of SCZFN against *A. sinensis* root rot compared to conventional fungicides.

The rhizosphere of plants functions as a specialized habitat for diverse microorganisms, which play direct roles in disease pathogenesis, prevention, and plant growth and health [32,36]. Soilborne pathogens typically derive nutrients from the soil or root secretions, enhancing their abundance in the soil and occupying the ecological niche of the soil microbial community to facilitate infection of plant roots [37,38]. Consequently, most studies on controlling soilborne diseases have focused on reducing the prevalence of pathogens [5,25]. Our analysis of microbial communities revealed that all three treatments decreased the relative abundance of the genus *Fusarium*, the primary pathogen responsible for *A. sinensis* root rot. Notably, T1 and T3 exhibited significantly more significant reductions compared to T2. This finding aligns with previous reports indicating that a novel attapulgite-coated biocontrol agent can effectively control root rot by diminishing the relative abundance of *Fusarium* [7]. From the perspective of reducing the abundance of the primary pathogen to control root rot occurrence effectively, the three treatments exhibited varying degrees of prevention against *A. sinensis* root rot by significantly decreasing *Fusarium* abundance and efficiently inhibiting the abundance of auxiliary pathogens of genus *Humicola* and *Chrysosporium*. Genus *Humicola* has been reported as the causal agent of *Pinus pinea* root rot, and it is also a prominent fungal genus associated with tomato root infestation, causing a similar disease characterized by root xylem rot; however, it does not directly initiate the disease [39,40]. *Chrysosporium* is a pathogen associated with white rot; however, no studies have reported that it can cause root rot [41,42]. However, variations in control effectiveness among the three treatments indicated that they also exerted diverse effects on other microorganisms within the rhizosphere soil microbiome.

Alternatively, pathogens can be prevented by introducing additional soil-beneficial microorganisms to occupy specific ecological niches or by stimulating plant resistance mechanisms [24,32,43]. Our microbiome analysis showed that T1 significantly increased the relative abundance of soil-beneficial microorganisms involved in carbon cycling promotion (*Acidobacteria*), nitrogen enrichment (*Nitrospira*), and organic matter turnover for soil health improvement (*Pedosphaeraceae*). Consistent with the results and views of Tao and colleagues, introducing beneficial microorganisms induces plant resistance and can protect plants from the infection of major pathogens [44]. Surprisingly, despite having comparable efficacy, T3 and T1 exhibited contrasting effects on the proliferation of these beneficial microorganisms, suggesting a non-discriminatory inhibition of all organisms. The findings of our study align with previous reports indicating that chemical pesticides, particularly those comprising complex constituents and exhibiting long-lasting properties, exhibit potent inhibitory effects against a diverse range of bacteria or fungi. Furthermore, it has been reported that the continuous application of chemical pesticides detrimentally influences soil microbial communities’ richness, diversity, composition, and functionality [45]. Many studies have demonstrated the influence of microbial community stability and diversity on plant growth and health [38,46]. Whether by enhancing soil microbial diversity [25] or establishing beneficial microbial flora [26,47] to suppress the proliferation of pathogenic bacteria for effective disease control.

The findings of our study are in general agreement with previous reports, indicating that agricultural soils predominantly comprise bacterial phyla Proteobacteria, followed by Acidobacteriota, Bacteroidota, and Gemmatimonadota dominated the soil bacterial communities [25,26]. Our study found that the RA of these phyla in T1, T2, T3, and CK were around 67.4%, 74.4%, 81.5%, and 74.8% respectively. Fungal phyla such as Ascomycota, Basidiomycota, and Mortierellomycota dominated the soil fungal communities and accounted for more than 99% RA in T1, T3, and CK except T2 [25,48]. The high-throughput sequencing results revealed significant effects of T1 and T2 treatments on the diversity and composition of microbial communities in rhizosphere soil. We observed significantly higher values of α diversity index (including Chao 1 index, Shannon index, and Pielou evenness index) of bacterial communities under T1 treatment. Moreover, the α diversity index of the fungal community was significantly higher under T2 treatment. These findings suggest that T1 effectively enhances the diversity of the rhizosphere bacterial community, consistent with our observation that it increases the abundance of beneficial bacterial genera *Acidobacteria*, *Nitrospira,* and *Pedosphaeraceae*. According to the previous research report, the bacterial community plays a pivotal role in carbon cycling [49] and biochar amendment, further enhancing plant carbon assimilation through modulation of rhizosphere bacterial communities for improved tomato growth [50]. Additionally, PCoA results showed that T1 and T3 were clustered separately, and T1 has a tighter degree of clustering, with a clear separation between CK compared to other treatments, indicating that bacterial and fungal community composition significantly differed under the application of SCZFN, which is similar to the results of previous reports [25,51].

The analysis of the intra-kingdom co-occurrence network revealed that the relationships between microorganisms became more intricate under T1 treatment. Although there was no difference in the number of nodes, the number of edges significantly increased compared to other treatments and CK, indicating higher network connectivity and complex microbial interactions. This suggests that T1 can enhance the recruitment or introduction of microorganisms by inhibiting pathogenic bacteria from occupying rhizosphere soil niches. These recruited microorganisms also play a crucial role in suppressing pathogens infections, promoting greater diversity within the microbial community, and ensuring a more stable and healthy structure for the bacterial–fungal interkingdom network than the CK network. Previous studies have reported that pathogen invasion significantly impacts the rhizosphere microbiome’s assembly, while the application of biological control agents alters the structure and composition of this microbial community [25,52,53]. Further analysis of the root rot incidence and microbial community based on the Pearson correlation coefficient revealed consistent results with our previous findings. Numerous studies have demonstrated the intricate nature of factors contributing to soilborne diseases, indicating that the infection process is not solely driven by a single major pathogen but rather involves multiple auxiliary pathogens in common co-infection [25,54,55]. Bacterial genera *Muribaculaceae* and fungal genera *Humicola*, *Fusarium,* and *Mycochlamys* were significantly associated with the disease incidence of root rot, and these microorganisms exhibited significant enrichment in the CK. The primary pathogen, *Fusarium*, has been extensively reported in numerous studies [4,5]. Therefore, we supposed that *Muribaculaceae* spp., *Humicola* spp., and *Mycochlamys* spp. act as assisting microorganisms that create a favorable environment for *Fusarium* to infect the root system of *A. sinensi*. However, further study is required to elucidate their mutualistic relationship in the occurrence of root rot disease.

In general, biopesticides not only affect rhizosphere soil microbial diversity but also affect plant metabolites’ types, accumulation, and metabolic pathways while preventing and controlling diseases [56]. Plant activates their protective mechanisms and regulates their metabolites and metabolic pathways in response to pathogens, including many plant-derived biopesticides, which are also derived from plant secondary metabolites with significant antibacterial effects [57]. Secondary metabolites of plants are essential components of defense mechanisms against pathogen attack and environmental stress [58]. Abiotic and biotic environments that constantly change significantly influence the synthesis and accumulation of plant secondary metabolites [59]. Therefore, plant metabolomics analysis can elucidate the alterations and accumulation of *A. sinensis* under diverse treatments (directly) and the shifts in the microbial community (indirectly), thereby aiding in comprehending the impact of biopesticide treatment on secondary metabolite types and metabolic pathways of *A. sinensis* and root decay infection on its respective counterparts. The results of this study revealed the identification of 799 metabolites with LC-MS approach. Lipids, lipid-like molecules, organic acids, and derivatives were the most abundant types, accounting for over 50% of all secondary metabolites (in both positive and negative ion modes), representing the primary type reported in studies on *A. sinensis* [60].

According to the PCA results, the metabolite types and accumulation of *A. sinensis* under different treatments were different, and the three treatments were also distinct from CK. This indicates a significant influence of *A. sinensis* on metabolite types and accumulation under various treatments. Additionally, root rot disease resulted in substantial changes in both types and accumulation of *A. sinensis* metabolites. This finding contradicts our initial conjecture that after effective control by T1 and T3 treatments, the type and accumulation of secondary metabolites would recover to similar levels. Consequently, this conclusion provides a clear explanation for plant disease occurrence as well as why chemical pesticide residues lead to significant differences in active component content within medicinal plants [61]. Based on these findings, we investigated the abundance of differentially expressed metabolites (DEMs) and their regulatory relationships under various treatments. It was observed that compared to the CK, the T2 treatment exhibited 105 DEMs, including 81 unique metabolites, and showed enrichment in 41 metabolic pathways, surpassing the other two treatments. However, no metabolic pathway was significantly enriched in the KEGG pathway (one pathway in T1 treatment and five in T3 treatment). This indicates that although T2 treatment has more effects on the types of metabolites of *A. sinensis*, it cannot profoundly affect the metabolic pathway of *A. sinensis*.

Plant-associated rhizosphere microbial communities play a pivotal role in the biosynthesis and accumulation of secondary metabolites in medicinal plants [62]. The rhizosphere serves as a crucial interface for plant-soil-microbial information and material exchange, with the interaction between plant roots and rhizosphere microorganisms being essential for optimal plant growth and quality [63,64]. Therefore, we analyzed and investigated the relationship between different rhizosphere microbial communities and DEMs under various treatments. The comparison of microbial communities and DEMs among T1, T3, and CK revealed a higher level of complexity and correlation. Additionally, the correlation between the top 20 differentially abundant microbial genera treated by T1 and DEMs exhibited significantly stronger associations. The noteworthy observation is that *Sphingobium*, *Dyadobacter*, and *Halomonas*, which have been found to be beneficial for plant and soil environments, exhibited significant associations with the majority of DEMs in the Top 30. The correlation analysis of T2 was consistent with the above conclusion, and there was no significant correlation between differential microorganisms and differential expression of metabolites. Treatment T3 showed complex correlations between the microbiome and differential metabolites, but the top 20 differential microbes were not significantly associated with the top 30 differential metabolites.

## 4. Materials and Methods

### 4.1. Site and Experimental Design Descriptions

The field experiment was conducted in the *Angelica sinensis* green standard planting area, located in Minxian County (34°36′ N, 104°00′ E), Dingxi City, Gansu Province, China, in 2022. *A. sinensis* has been continuously cultivated in the field for more than 10 years, and the field is seriously infected with root rot disease. The climate is cold and wet, with an average annual temperature of 6.1 °C and average annual precipitation of about 650 mm. The soil was thoroughly turned and mixed with insecticide 3% phoxim granules prior to transplanting *A. sinensis* seedlings to kill the larvae of insect pests. The experiment was performed under four distinct treatments: T1; application of biopesticide Shi Chuang Zhi Feng Ning (SCZFN), T2; application of *Bacillus subtilis* wettable powder (manufactured by the GenTaiKeAn Company, Shandong, China), T3; application of pesticide Apron Advance (thiabendazole·fludioxonil·metalaxyl-M 18% FSC; Syngenta, Shanghai, China), commonly used by farmers, and water treatment was implemented as a control (CK). Three months after seedling transplantation, throughout the growth period of *A. sinensis*, all treatments were applied four times at one-month intervals. All field management approaches throughout the entire growth period of *A. sinensis* were carried out according to national standards except for disease control [7]. Seedlings of *A. sinensis* were transplanted in a plot (5 × 5 m^2^) with a plant-to-row spacing of 40 cm × 40 cm, and the experiment was conducted under a randomized complete block design. A row spacing area of 1 m wide was established between each plot. The experiment was performed in replicates with three plots per treatment as biological replicates and 140 seedlings per plot.

### 4.2. Samples Collections and Disease Severity Analysis

Rhizosphere soil and *A. sinensis* root samples were collected from each plot upon observation of *Fusarium* root rot symptoms. Sixty plants were randomly selected using an S-type sampling method [65] from each treatment (15 plants per plot) and transported to the laboratory in dry ice. The rhizosphere soil adhering to the roots (10 plants per replication) was collected precisely and mixed to make one composite sample to analyze the rhizosphere microbiome [26]. Meanwhile, the same *A. sinensis* roots (10 roots per replication) were crushed and mixed to make one sample for a metabolomic study. Briefly, 2 g of mixed rhizosphere soil and crushed root samples from each replication were collected in 5 mL of sterilized falcon tubes, followed by rapid freezing in liquid nitrogen and stored at −80 °C for subsequent studies. The efficacy of each treatment group was assessed using the disease index method based on the percentage of root lesion area according to the disease classification scale (0, 1, 2, 3, 4, and 5) [7]. Here, no or few diseases spot (0), the infected area is not higher than 10% of the whole root area (1), the infected area accounted for 11–30% of the whole root area (2), the infected area accounted for 31–50% of the whole root area (3), the infected area accounted for more than 50% of the whole root area (4), and the infected area accounted for more than 75% of the whole root area and the plant died (5). The disease incidence (Di), disease index (DI), and control effect (C.E) were calculated using the following formulas: Di (%) = (no. of infected plants/total no. of plants) × 100, DI = sum ((disease ratings scale × no. of infected plants in each index)/(total number of plants × maximum disease rating scale)). C.E (%) = (DI of control − DI of treatment) × 100/ DI of control.

### 4.3. Soil DNA Extraction, PCR Amplification, and Amplicon Sequencing

Genomic DNA from soil was extracted using a PowerSoil DNA Isolation Kit (MoBio Laboratories, Carlsbad, CA, USA) following the instructions provided by the manufacturer. The purity and concentration of extracted DNA were assessed using an ND2000 spectrophotometer and diluted (1 ng/µL) using sterilized water if required. PCR was amplified for the V4 region of the 16S rRNA gene of bacteria [66] and the ITS1-5F region of the ITS gene of fungi [67]. The sequence libraries were prepared according to Illumina HiSeq protocols and were sequenced on an Illumina HiSeq platform to produce paired-end reads of 250 bp by Novogene Co., Ltd. (Shanghai, China).

### 4.4. Microbiome Data Processing and Bioinformatics Analysis

Quality control of raw reads was performed by fastp (Version 0.20.0) [68], and chimera sequences were deleted using Vsearch (Version 2.15.0) to get clean reads [69]. The sequencing and species annotation of clean reads were conducted using DADA2 (v. 1.32.0) and QIIME2 software (QIIME 2 2023.5) to produce the amplicon sequence variants (ASVs), and ASVs with an abundance of less than 5 were excluded [70,71]. Silva database (https://www.arbsilva.de) was used for bacterial taxonomic annotation, and the fungi unite database (https://unite.ut.ee/) was used [72,73]. The alpha diversity indices (Chao 1, Shannon, Simpson, and Pielou evenness) and beta diversity based on the Bray–Curtis distance matrix were calculated using QIIME 2 [71] and visualized using boxplots and principal coordinate analysis (PCoA) analysis, respectively, using the ggplot2 package in R software (version 4.4.0), and PERMANOVA based on Adonis was performed to calculate overall variations in the microbial communities [74]. The relative abundance bar plots and violin box diagram at phylum for ASVs were generated using GraphPad Prism (v 9.0.0), and the chord diagram was made using Circos Table Viewer (v0.63-10) online (https://mk.bcgsc.ca/tableviewer/visualize/). Significant differences among microbial communities were calculated using the Wilcoxon test at *p* < 0.05. We used sparCC in R to analyze microbial co-occurrence networks for bacterial and fungal communities in ASVs at the genus level (*p* < 0.05 and correlation coefficient > 0.3) [25] and were visualized in Gephi 0.9.2. Correlation analysis was achieved according to the Pearson correlation coefficient (*p* < 0.05) between disease incidence and bacterial–fungal genera and using an online bioinformatics platform (https://www.bioinformatics.com.cn).

### 4.5. Metabolites UHPLC-MS/MS Analysis

For metabolomic analysis, 100 mg of root tissues (as collected above) were grounded with liquid nitrogen, and the homogenate was resuspended with prechilled 80% methanol by well vortex [75]. The samples were incubated on ice for 5 min and centrifuged at 15,000× *g*, 4 °C for 20 min. The supernatant was diluted to a final concentration containing 53% methanol by LC-MS grade water. The samples were transferred to a fresh Eppendorf tube and centrifuged at 15,000× *g*, 4 °C for 20 min. Finally, the supernatant was injected into the LC-MS/MS system analysis. UHPLC-MS/MS analyses were performed using a Vanquish UHPLC system (ThermoFisher, Germany) coupled with an Orbitrap Q Exactive^TM^HF-X mass spectrometer (Thermo Fisher, Dreieich, Germany) by Novogene Co., Ltd. (Beijing, China). Samples were injected onto a Hypesil Gold column (100 × 2.1 mm, 1.9 μm) using a 17-min linear gradient at a 0.2 mL/min flow rate. The eluents for the positive polarity mode were eluent A (0.1% FA in water) and eluent B (methanol). The eluents for the negative polarity mode were eluent A (5 mM ammonium acetate, pH 9.0) and eluent B (methanol). The solvent gradient was set as follows: 2% B, 1.5 min; 2–85% B, 3 min; 85–100% B, 10 min; 100–2% B, 10.1 min; 2% B, 12 min. Q Exactive^TM^ HF-X mass spectrometer was operated in positive/negative polarity mode with a spray voltage of 3.5 kV, capillary temperature of 320 °C, sheath gas flow rate of 35 psi, and aux gas flow rate of 10 L/min, S-lens RF level of 60, Aux gas heater temperature of 350 °C.

### 4.6. Data Processing and Metabolite Identification

The raw data files generated by UHPLC-MS/MS were processed using the Compound Discoverer 3.1 (CD3.1, Thermo Fisher Dreieich, Germany) to perform peak alignment, peak picking, and quantitation for each metabolite under positive ion mode (POS) and negative ion mode (NEG) [76]. The normalized data was used to predict the molecular formula based on additive ions, molecular ion peaks, and fragment ions. Then, the peaks were matched with the mzCloud (https://www.mzcloud.org/), mzVault, and MassList databases to obtain accurate qualitative and relative quantitative results [77]. Statistical analyses were performed using the statistical software R (v3.5.0), and compounds whose CVs of relative peak areas in QC samples were greater than 30% were removed for metabolite identification and relative quantification.

### 4.7. Metabolomics Data Analysis

These metabolites were annotated using the KEGG database (https://www.genome.jp/kegg/pathway.html), HMDB database (https://hmdb.ca/metabolites), and LIPIDMaps database (http://www.lipidmaps.org/). The centering and scaling of metabolome raw data were performed using the R scale function in R. Principal components analysis (PCA), and partial least squares discriminant analysis (PLS-DA) was performed using metaX [78], and univariate analysis (*t*-test) was performed to calculate the statistical significance (*p*-value). The metabolites with VIP > 1, *p* < 0.05, fold change (FC) ≥ 2, or ≤0.5 were assigned as differentially expressed metabolites (DEMs) [79]. Volcano plots were used to filter metabolites of interest based on log_2_FC and −log_10_(*p*-value) of metabolites by “ggplot2” in R (v3.5.0). For hierarchical clustering analysis (HCA), the data were normalized using z-scores of the intensity areas of differential metabolites and were visualized by “Pheatmap” package in R. The functions of these metabolites and metabolic pathways were studied using the KEGG database. The metabolic pathway enrichment of differential metabolites was performed; when the ratio was satisfied by x/n > y/N, the metabolic pathway was considered as enriched and when the *p*-value of the metabolic pathway < 0.05, the metabolic pathway was considered as statistically significant enrichment.

### 4.8. Correlations Analysis between Differential Microorganisms and DEMs and Statistical Analysis

Using the linkET package in R (v3.5.0), the Mantel test was used to investigate Pearson’s r linkages between root secondary metabolites and rhizosphere microorganisms [64]. Correlation analysis of differential rhizosphere microorganisms (bacterial–fungal genera) in the top twenty and differential expressed metabolites in the top thirty (POS and NEG) was performed using the OmicShare tool, a free online platform for data analysis (https://www.omicshare.com/tools). IBM SPSS Version 20.0 (SPSS Inc., Chicago, IL, USA) was used to calculate the significant differences among treatments according to Duncan’s multiple range test at *p* < 0.05 based on analysis of variance (ANOVA). All figures were adjusted, combined, and modified using Adobe Illustrator 2019.

## 5. Conclusions

In conclusion, the application of biopesticide SCZNF significantly mitigates root rot incidence in *Angelica sinensis* by modulating the host microbiome and root metabolites. Application of SCZNF (T1) showed a control effect of 73.17%, similar to fungicide (T2) 75.45%, significantly higher than *Bacillus subtilis* (T2; 39.99%). This effectiveness is attributed to T1 and T3 ability to lower the abundance of fungal genera, including *Fusarium* spp., *Humicola* spp., and *Chrysosporium* spp., known to cause root rot disease and continuous cropping issues. Treatment T1 significantly enhanced the abundance of beneficial bacterial taxa such as *Acidobacteria* spp., *Nitrospira* spp., and *Pedosphaeraceae* spp., which promote plant growth and soil health through improved carbon cycling and nitrogen enrichment. Additionally, T1 fostered a more complex and stable interkingdom network, indicating better recruitment of beneficial microbes and suppression of root rot pathogens. Metabolomic analysis showed that all treatments significantly affected the secondary metabolites in *A. sinensis* roots, with T2 leading to a greater enrichment of metabolic pathways and 105 differentially expressed metabolites compared to the control. However, correlation analysis revealed a stronger association between microbial communities and metabolites in T1 and T3 than in T2. Overall, this study underscores the intricate interactions between *A. sinensis*, its root metabolome, and the rhizosphere microbial community in influencing disease susceptibility.

## Figures and Tables

**Table 1:** The assessment of the control efficacy of various treatments on *Angelica sinensis* root rot disease occurrence.

Treatments	Disease Index (%)	Disease Incidence (%)	Control Effect (%)
CK	73.33 a	92.00 a	----- c
T1	19.67 c	28.33 c	73.17 a
T2	44.00 b	47.83 b	39.99 b
T3	18.00 c	23.61 c	75.45 a

CK; water treatment as a control (CK), T1; application of biopesticide shi chuang zhi feng ning (SCZFN), T2; application of *Bacillus subtilis* wettable powder, and T3; application of fungicide Apron Advance (Swiss, Syngenta). Different lowercase letters within the column show significant differences among treatments according to Duncan’s multiple range test at *p* < 0.05.

## Data Availability

All raw data related to 16S and ITS amplicon sequencing have been submitted to the public database NCBI and are available as a Sequence Read Archive (SRA) under BioProject No. PRJNA1119185. https://www.ncbi.nlm.nih.gov/bioproject/?term=No.%20PRJNA1119185.

## Supplementary Materials

The following supporting information can be downloaded at: https://www.mdpi.com/article/10.3390/plants13162180/s1. Figure S1. The systematic tree of representative sequences of species within the genus. Figure S2. Pearson correlation coefficient between QC samples based on the relative quantitative values of metabolites. Figure S3. Pie chart of metabolite classification. Figure S4. Principal component analysis was conducted on metabolites of different treatments and the metabolites that passed quality control. Figure S5. Partial least-squares discrimination analysis (PLS-DA) of different metabolites under different treatments. Figure S6. Function and classification of metabolites in three databases. Figure S7. Hierarchical clustering analysis of all differentially expressed metabolites between different treatments. Table S1. Statistical table of 16S sequencing data. Table S2. Statistical table of ITS sequencing data. Table S3. The top 10 taxonomy relative abundance in bacterial phyla. Table S4. The top 10 taxonomy relative abundance in fungal phyla. Table S5. Metabolite differential screening results.

## References

[1] Han Y., Chen Y., Zhang Q., Liu B.-W., Yang L., Xu Y.-H., Zhao Y.-H. (2021). Overview of therapeutic potentiality of *Angelica sinensis* for ischemic stroke. Phytomedicine.

[2] Cai Y., Wang Y., Su W., Zhou X., Lu C. (2024). Angelica sinensis polysaccharide suppresses the Wnt/β-catenin-mediated malignant biological behaviors of breast cancer cells via the miR-3187-3p/PCDH10 axis. Biochem. Pharmacol..

[3] Shen X., Wu Y., Chen P., Bai Y., Liu Y., Jiang Y., Zhang Y., Yang Z. (2024). Anti-platelet aggregation activities of different grades of Angelica sinensis and their therapeutic mechanisms in rats with blood deficiency: Insights from metabolomics and lipidomics analyses. Front. Pharmacol..

[4] Zhang Z., Zhang W., Wang X., Kou Z., Wang Y., Islam R., Zhang J., Liu L., Shen T., Tian Y. (2023). Isolation and identification of antagonistic bacteria of *Angelica* root rot and their mechanism as biological control. Biol. Control.

[5] Liu Y., Tian Y., Zhao X., Yue L., Uwaremwe C., Zhou Q., Wang Y., Zhang Y., Dun Z., Cui Z. (2022). Identification of pathogenic Fusarium spp. responsible for root rot of *Angelica sinensis* and characterization of their biological enemies in Dingxi, China. Plant Dis..

[6] Zhang Y., Wang R., Xie Z., Wang Y., Zhao X., Liu Y., Zhou Q. (2021). Rapid visual detection of Japanese hornwort mosaic virus infecting *Angelica sinensis* by reverse transcription loop-mediated isothermal amplification. Ann. Appl. Biol..

[7] Liu Y., Tian Y., Yue L., Constantine U., Zhao X., Zhou Q., Wang Y., Zhang Y., Chen G., Dun Z. (2021). Effectively controlling Fusarium root rot disease of *Angelica sinensis* and enhancing soil fertility with a novel attapulgite-coated biocontrol agent. Appl. Soil Ecol..

[8] Uwaremwe C., Yue L., Liu Y., Tian Y., Zhao X., Wang Y., Xie Z., Zhang Y., Cui Z., Wang R. (2021). Molecular identification and pathogenicity of *Fusarium* and *Alternaria* species associated with root rot disease of wolfberry in Gansu and Ningxia provinces, China. Plant Pathol..

[9] Zhu B., Wu J., Ji Q., Wu W., Dong S., Yu J., Zhang Q., Qin L. (2020). Diversity of rhizosphere and endophytic fungi in *Atractylodes macrocephala* during continuous cropping. PeerJ.

[10] Ma H., Duan X., Xu W., Ma G., Ma W., Qi H. (2022). Root Rot of *Angelica sinensis* Caused by Clonostachys rosea and Fusarium acuminatum in China. Plant Dis..

[11] Farh M.E.-A., Kim Y.-J., Kim Y.-J., Yang D.-C. (2018). *Cylindrocarpon destructans/Ilyonectria radicicola*-species complex: Causative agent of ginseng root-rot disease and rusty symptoms. J. Ginseng Res..

[12] Wang L., Xi N., Lang D., Zhou L., Zhang Y., Zhang X. (2022). Potential biocontrol and plant growth promotion of an endophytic bacteria isolated from *Glycyrrhiza uralensis* seeds. Egypt. J. Biol. Pest Control.

[13] Zhang C., Liu Z., Yang Y., Ma Q., Zheng Y., Xu C., Gao X., Gao W., Huang Z., Liu X. (2024). Characterization of *Fusarium* species causing soybean root rot in Heilongjiang, China, and mechanism underlying the differences in sensitivity to DMI fungicides. Pestic. Biochem. Physiol..

[14] Myresiotis C.K., Karaoglanidis G.S., Vryzas Z., Papadopoulou-Mourkidou E. (2012). Evaluation of plant-growth-promoting rhizobacteria, acibenzolar-S-methyl and hymexazol for integrated control of *Fusarium* crown and root rot on tomato. Pest Manag. Sci..

[15] Naseri B. (2014). Bean production and *Fusarium* root rot in diverse soil environments in Iran. J. Soil Sci. Plant Nutr..

[16] Liu Q., Yang J., Ahmed W., Wan X., Wei L., Ji G. (2022). Exploiting the antibacterial mechanism of phenazine substances from *Lysobacter antibioticus* 13-6 against *Xanthomonas oryzae* pv. oryzicola. J. Microbiol..

[17] Ahmed W., Yang J., Tan Y., Munir S., Liu Q., Zhang J., Ji G., Zhao Z. (2022). *Ralstonia solanacearum*, a deadly pathogen: Revisiting the bacterial wilt biocontrol practices in tobacco and other Solanaceae. Rhizosphere.

[18] Shen T., Wang C., Yang H., Deng Z., Wang S., Shen B., Shen Q. (2016). Identification, solid-state fermentation and biocontrol effects of *Streptomyces hygroscopicus* B04 on strawberry root rot. Appl. Soil Ecol..

[19] Kalantari S., Marefat A., Naseri B., Hemmati R. (2018). Improvement of bean yield and *Fusarium* root rot biocontrol using mixtures of *Bacillus, Pseudomonas* and *Rhizobium*. Trop. Plant Pathol..

[20] Zhang J., Wei L., Yang J., Ahmed W., Wang Y., Fu L., Ji G. (2020). Probiotic consortia: Reshaping the rhizospheric microbiome and its role in suppressing root-rot disease of *Panax notoginseng*. Front. Microbiol..

[21] Zhang J., Ahmed W., Dai Z., Zhou X., He Z., Wei L., Ji G. (2022). Microbial consortia: An engineering tool to suppress clubroot of Chinese cabbage by changing the rhizosphere bacterial community composition. Biology.

[22] Liu Y., Burke D.J., Medeiros J.S., Carrino-Kyker S.R., Burns J.H. (2023). Phosphite indirectly mediates protection against root rot disease via altering soil fungal community in *Rhododendron* species. Plant Soil.

[23] Zhang M., Kong Z., Fu H., Shu X., Xue Q., Lai H., Guo Q. (2023). Rhizosphere microbial ecological characteristics of strawberry root rot. Front. Microbiol..

[24] Ling N., Wang T., Kuzyakov Y. (2022). Rhizosphere bacteriome structure and functions. Nat. Commun..

[25] Ahmed W., Dai Z., Zhang J., Li S., Ahmed A., Munir S., Liu Q., Tan Y., Ji G., Zhao Z. (2022). Plant-microbe interaction: Mining the impact of native *Bacillus amyloliquefaciens* WS-10 on tobacco bacterial wilt disease and rhizosphere microbial communities. Microbiol. Spectr..

[26] Dai Z., Ahmed W., Yang J., Yao X., Zhang J., Wei L., Ji G. (2023). Seed coat treatment by plant-growth-promoting rhizobacteria *Lysobacter antibioticus* 13–6 enhances maize yield and changes rhizosphere bacterial communities. Biol. Fertil. Soils.

[27] Chaloner T.M., Gurr S.J., Bebber D.P. (2020). Geometry and evolution of the ecological niche in plant-associated microbes. Nat. Commun..

[28] Qu Q., Li Y., Zhang Z., Cui H., Zhao Q., Liu W., Lu T., Qian H. (2021). Effects of S-metolachlor on wheat (*Triticum aestivum* L.) seedling root exudates and the rhizosphere microbiome. J. Hazard. Mater..

[29] Chen J.-M., Feng W.-M., Yan H., Liu P., Zhou G.-S., Guo S., Yu G., Duan J.-A. (2022). Explore the interaction between root metabolism and rhizosphere microbiota during the growth of *Angelica sinensis*. Front. Plant Sci..

[30] Musilova L., Ridl J., Polivkova M., Macek T., Uhlik O. (2016). Effects of secondary plant metabolites on microbial populations: Changes in community structure and metabolic activity in contaminated environments. Int. J. Mol. Sci..

[31] Wen T., Zhao M., Yuan J., Kowalchuk G.A., Shen Q. (2021). Root exudates mediate plant defense against foliar pathogens by recruiting beneficial microbes. Soil Ecol. Lett..

[32] Liu H., Li J., Carvalhais L.C., Percy C.D., Prakash Verma J., Schenk P.M., Singh B.K. (2021). Evidence for the plant recruitment of beneficial microbes to suppress soil-borne pathogens. New Phytol..

[33] Al-Khayri J.M., Rashmi R., Toppo V., Chole P.B., Banadka A., Sudheer W.N., Nagella P., Shehata W.F., Al-Mssallem M.Q., Alessa F.M. (2023). Plant secondary metabolites: The weapons for biotic stress management. Metabolites.

[34] Yeshi K., Crayn D., Ritmejerytė E., Wangchuk P. (2022). Plant secondary metabolites produced in response to abiotic stresses has potential application in pharmaceutical product development. Molecules.

[35] Carezzano M.E., Reyna P.G., Accotto E., Giordano W., Oliva M.d.l.M., Rodriguez Pardina P., Sabini M.C. (2023). Plant-Derived Essential Oils and Aqueous Extract as Potential Ingredients for a Biopesticide: Phytotoxicity in Soybean and Activity against Soybean Mosaic Virus. Processes.

[36] Thepbandit W., Athinuwat D. (2024). Rhizosphere Microorganisms Supply Availability of Soil Nutrients and Induce Plant Defense. Microorganisms.

[37] Fatima U., Senthil-Kumar M. (2015). Plant and pathogen nutrient acquisition strategies. Front. Plant Sci..

[38] Liu L., Huang X., Zhang J., Cai Z., Jiang K., Chang Y. (2020). Deciphering the relative importance of soil and plant traits on the development of rhizosphere microbial communities. Soil Biol. Biochem..

[39] Ginetti B., Uccello A., Bracalini M., Ragazzi A., Jung T., Moricca S. (2012). Root rot and dieback of *Pinus pinea* caused by phytophthora Humicola in Tuscany, central Italy. Plant Dis..

[40] Menzies J.G., Ehret D.L., Koch C., Bogdanoff C. (1998). *Humicola fuscoatra* infects tomato roots, but is not pathogenic. Eur. J. Plant Pathol..

[41] Zhang X., Shao J., Chen A., Shang C., Hu X., Luo S., Lei M., Peng L., Zeng Q. (2018). Effects of cadmium on calcium homeostasis in the white-rot fungus *Phanerochaete chrysosporium*. Ecotoxicol. Environ. Saf..

[42] Xie J., Ming Z., Li H., Yang H., Yu B., Wu R., Liu X., Bai Y., Yang S.-T. (2016). Toxicity of graphene oxide to white rot fungus *Phanerochaete chrysosporium*. Chemosphere.

[43] Vallance J., Déniel F., Floch G.L., Guérin-Dubrana L., Blancard D., Rey P. (2011). Pathogenic and beneficial microorganisms in soilless cultures. Sustain. Agric..

[44] Tao C., Wang Z., Liu S., Lv N., Deng X., Xiong W., Shen Z., Zhang N., Geisen S., Li R. (2023). Additive fungal interactions drive biocontrol of *Fusarium* wilt disease. New Phytol..

[45] Shahid M., Khan M.S. (2022). Ecotoxicological implications of residual pesticides to beneficial soil bacteria: A review. Pestic. Biochem. Physiol..

[46] Yang H., Li J., Xiao Y., Gu Y., Liu H., Liang Y., Liu X., Hu J., Meng D., Yin H. (2017). An integrated insight into the relationship between soil microbial community and tobacco bacterial wilt disease. Front. Microbiol..

[47] Martins S.J., Pasche J., Silva H.A.O., Selten G., Savastano N., Abreu L.M., Bais H.P., Garrett K.A., Kraisitudomsook N., Pieterse C.M. (2023). The use of synthetic microbial communities to improve plant health. Phytopathology®.

[48] Bai Y.-C., Li B.-X., Xu C.-Y., Raza M., Wang Q., Wang Q.-Z., Fu Y.-N., Hu J.-Y., Imoulan A., Hussain M. (2022). Intercropping walnut and tea: Effects on soil nutrients, enzyme activity, and microbial communities. Front. Microbiol..

[49] Staley C., Ferrieri A.P., Tfaily M.M., Cui Y., Chu R.K., Wang P., Shaw J.B., Ansong C.K., Brewer H., Norbeck A.D. (2017). Diurnal cycling of rhizosphere bacterial communities is associated with shifts in carbon metabolism. Microbiome.

[50] Liao H., Zheng C., Long J., Guzmán I. (2021). Effects of biochar amendment on tomato rhizosphere bacterial communities and their utilization of plant-derived carbon in a calcareous soil. Geoderma.

[51] Hu Y., Zhao W., Li X., Feng J., Li C., Yang X., Guo Q., Wang L., Chen S., Li Y. (2021). Integrated biocontrol of tobacco bacterial wilt by antagonistic bacteria and marigold. Sci. Rep..

[52] Wei Z., Hu J., Yin S., Xu Y., Jousset A., Shen Q., Friman V.-P. (2018). *Ralstonia solanacearum* pathogen disrupts bacterial rhizosphere microbiome during an invasion. Soil Biol. Biochem..

[53] Bakker P.A., Doornbos R.F., Zamioudis C., Berendsen R.L., Pieterse C.M. (2013). Induced systemic resistance and the rhizosphere microbiome. Plant Pathol. J..

[54] Li M., Pommier T., Yin Y., Wang J., Gu S., Jousset A., Keuskamp J., Wang H., Wei Z., Xu Y. (2022). Indirect reduction *of Ralstonia solanacearum* via pathogen helper inhibition. ISME J..

[55] Venkatesh N., Koss M.J., Greco C., Nickles G., Wiemann P., Keller N.P. (2021). Secreted secondary metabolites reduce bacterial wilt severity of tomato in bacterial–fungal co-infections. Microorganisms.

[56] Ebrahimi-Zarandi M., Saberi Riseh R., Tarkka M.T. (2022). Actinobacteria as effective biocontrol agents against plant pathogens, an overview on their role in eliciting plant defense. Microorganisms.

[57] Dubrovina A., Kiselev K. (2017). Regulation of stilbene biosynthesis in plants. Planta.

[58] Divekar P.A., Narayana S., Divekar B.A., Kumar R., Gadratagi B.G., Ray A., Singh A.K., Rani V., Singh V., Singh A.K. (2022). Plant secondary metabolites as defense tools against herbivores for sustainable crop protection. Int. J. Mol. Sci..

[59] Verma N., Shukla S. (2015). Impact of various factors responsible for fluctuation in plant secondary metabolites. J. Appl. Res. Med. Aromat. Plants.

[60] Li Q., Chen Y., Gao H., Li Z., Qiu D., Hu G. (2023). In situ analysis of volatile oil in *Angelica sinensis* roots by fluorescence imaging combined with mass spectrometry imaging. Talanta.

[61] Kumar N., Kulsoom M., Shukla V., Kumar D., Priyanka, Kumar S., Tiwari J., Dwivedi N. (2018). Profiling of heavy metal and pesticide residues in medicinal plants. Environ. Sci. Pollut. Res..

[62] Jamwal V.L., Rather I.A., Ahmed S., Kumar A., Gandhi S.G. (2023). Changing Rhizosphere Microbial Community and Metabolites with Developmental Stages of Coleus barbatus. Microorganisms.

[63] Bhuyan B., Debnath S., Pandey P. (2020). The rhizosphere microbiome and its role in plant growth in stressed conditions. Rhizosphere Microbes: Soil and Plant Functions.

[64] Dang H., Zhang T., Wang Z., Li G., Zhao W., Lv X., Zhuang L. (2021). Succession of endophytic fungi and arbuscular mycorrhizal fungi associated with the growth of plant and their correlation with secondary metabolites in the roots of plants. BMC Plant Biol..

[65] Zhang J., Ahmed W., Zhou X., Yao B., He Z., Qiu Y., Wei F., He Y., Wei L., Ji G. (2022). Crop rotation with marigold promotes soil bacterial structure to assist in mitigating clubroot Incidence in Chinese Cabbage. Plants.

[66] Walters W., Hyde E.R., Berg-Lyons D., Ackermann G., Humphrey G., Parada A., Gilbert J.A., Jansson J.K., Caporaso J.G., Fuhrman J.A. (2016). Improved bacterial 16S rRNA gene (V4 and V4-5) and fungal internal transcribed spacer marker gene primers for microbial community surveys. Msystems.

[67] Yang Y., Hu J., Wei X., Huang K., Li C., Yang G. (2024). Deciphering core microbiota in rhizosphere soil and roots of healthy and Rhizoctonia solani-infected potato plants from various locations. Front. Microbiol..

[68] Haas B.J., Gevers D., Earl A.M., Feldgarden M., Ward D.V., Giannoukos G., Ciulla D., Tabbaa D., Highlander S.K., Sodergren E. (2011). Chimeric 16S rRNA sequence formation and detection in Sanger and 454-pyrosequenced PCR amplicons. Genome Res..

[69] Rognes T., Flouri T., Nichols B., Quince C., Mahé F. (2016). VSEARCH: A versatile open source tool for metagenomics. PeerJ.

[70] Callahan B.J., McMurdie P.J., Rosen M.J., Han A.W., Johnson A.J.A., Holmes S.P. (2016). DADA2: High-resolution sample inference from Illumina amplicon data. Nat. Methods.

[71] Bolyen E., Rideout J.R., Dillon M.R., Bokulich N.A., Abnet C.C., Al-Ghalith G.A., Alexander H., Alm E.J., Arumugam M., Asnicar F. (2019). Reproducible, interactive, scalable and extensible microbiome data science using QIIME 2. Nat. Biotechnol..

[72] Pruesse E., Quast C., Knittel K., Fuchs B.M., Ludwig W., Peplies J., Glöckner F.O. (2007). SILVA: A comprehensive online resource for quality checked and aligned ribosomal RNA sequence data compatible with ARB. Nucleic Acids Res..

[73] Kõljalg U., Larsson K.H., Abarenkov K., Nilsson R.H., Alexander I.J., Eberhardt U., Erland S., Høiland K., Kjøller R., Larsson E. (2005). UNITE: A database providing web-based methods for the molecular identification of ectomycorrhizal fungi. New Phytol..

[74] Zhang J., Zhou X., Zhang Y., Dai Z., He Z., Qiu Y., Alharbi S.A., Wei F., Wei L., Ahmed W. (2024). Pre-soil fumigation with ammonium bicarbonate and lime modulates the rhizosphere microbiome to mitigate clubroot disease in Chinese cabbage. Front. Microbiol..

[75] Li S., Deng B., Tian S., Guo M., Liu H., Zhao X. (2021). Metabolic and transcriptomic analyses reveal different metabolite biosynthesis profiles between leaf buds and mature leaves in Ziziphus jujuba mill. Food Chem..

[76] Cooper B., Yang R. (2024). An assessment of AcquireX and Compound Discoverer software 3.3 for non-targeted metabolomics. Sci. Rep..

[77] Ding Y., Chen S., Wang H., Li S., Ma C., Wang J., Cui L. (2021). Identification of secondary metabolites in Flammulina velutipes by UPLC-Q-Exactive-Orbitrap MS. J. Food Qual..

[78] Wen B., Mei Z., Zeng C., Liu S. (2017). metaX: A flexible and comprehensive software for processing metabolomics data. BMC Bioinform..

[79] Wei L., Yang J., Ahmed W., Xiong X., Liu Q., Huang Q., Ji G. (2021). Unraveling the association between metabolic changes in inter-genus and intra-genus bacteria to mitigate clubroot disease of Chinese cabbage. Agronomy.

