# Supplementary material for: A Novel Plant-Derived Biopesticide Mitigates Fusarium Root Rot of Angelica sinensis by Modulating the Rhizosphere Microbiome and Root Metabolome
Source: Plants (Basel). 2024 Aug 6;13(16):2180. doi: 10.3390/plants13162180 (PMC11360690; doi:10.3390/plants13162180)
Supplement: Supplementary file 1 [file plants-13-02180-s001.zip › plants-3086890-supplementary.pdf]

Article

A Novel Plant-Derived Biopesticide Mitigates *Fusarium* Root Rot of *Angelica sinensis* by Modulating the Rhizosphere Microbiome and Root Metabolome

Qi Liu [1,†], Waqar Ahmed [2,†], Guoli Li [1], Yilin He [1], Mohamed Mohany [3], Zhaoyu Li [1] and Tong Shen [1,*]

[1] Research Institute, Lanzhou Jiaotong University, Lanzhou 730070, China; liuqi@lzjtu.edu.cn (Q.L.); ligl@lzjtu.edu.cn (G.L.); heyl@lzjtu.edu.cn (Y.H.); lizy@mail.lzjtu.cn (Z.L.)
[2] Guangdong Province Key Laboratory of Microbial Signals and Disease Control, College of Plant Protection, South China Agricultural University, Guangzhou 510642, China; ahmed.waqar1083@yahoo.com
[3] Department of Pharmacology and Toxicology, College of Pharmacy, King Saud University, Riyadh 11451, Saudi Arabia; mmohany@ksu.edu.sa
* Correspondence: s_tong28@163.com
† These authors contributed equally to this work.

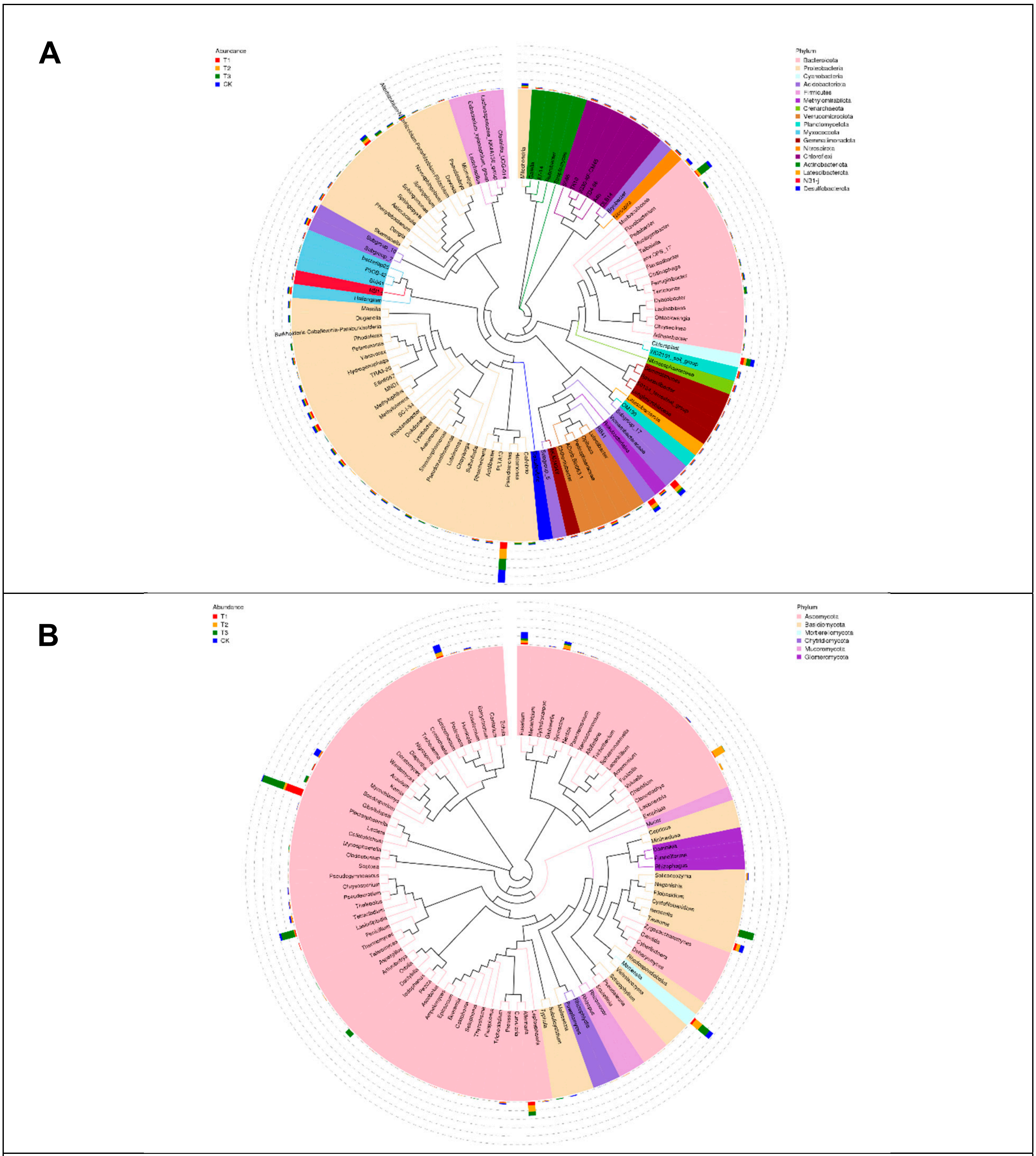

Figure S1. The systematic tree of representative sequences of species within the genus. Branches and fan shapes colored to represent the corresponding phylum, and the stacked bar chart outside the ring showing the distribution of abundance information of the genus in different samples. Bacteria (A) and fungi (B).

A

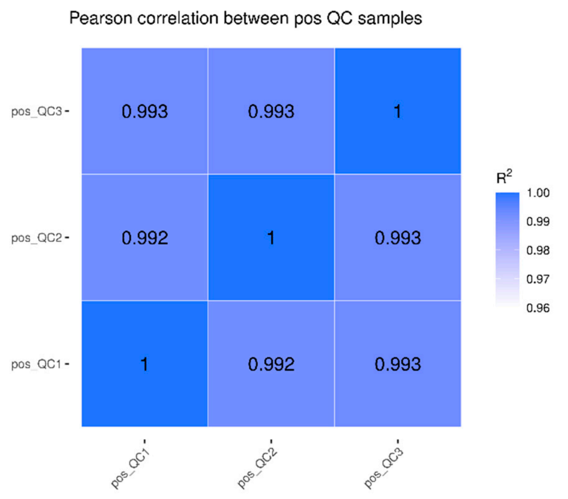

B

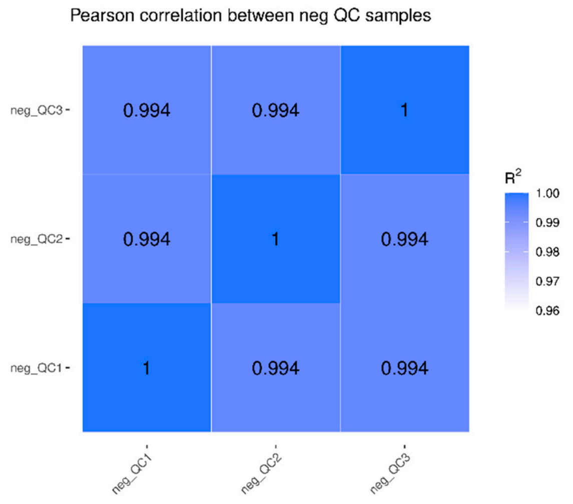

Figure S2. Pearson correlation coefficient between QC samples based on the relative quantitative values of metabolites. In positive mode (A) and in negative mode (B).

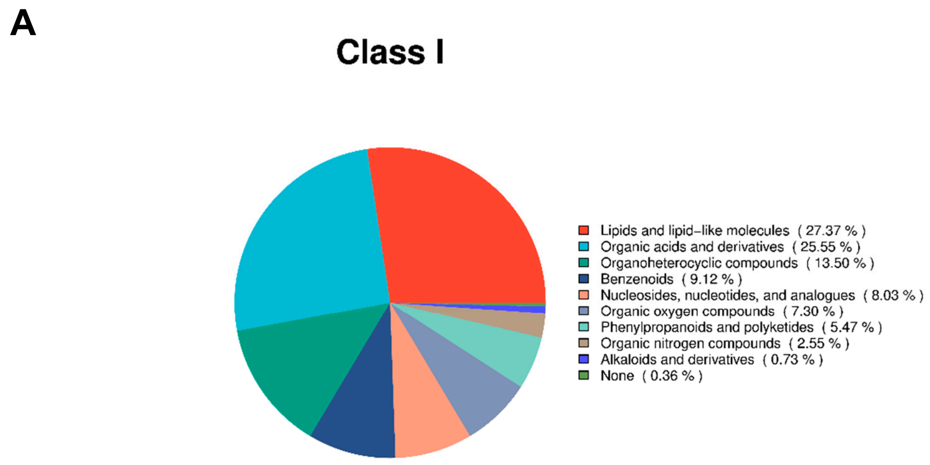

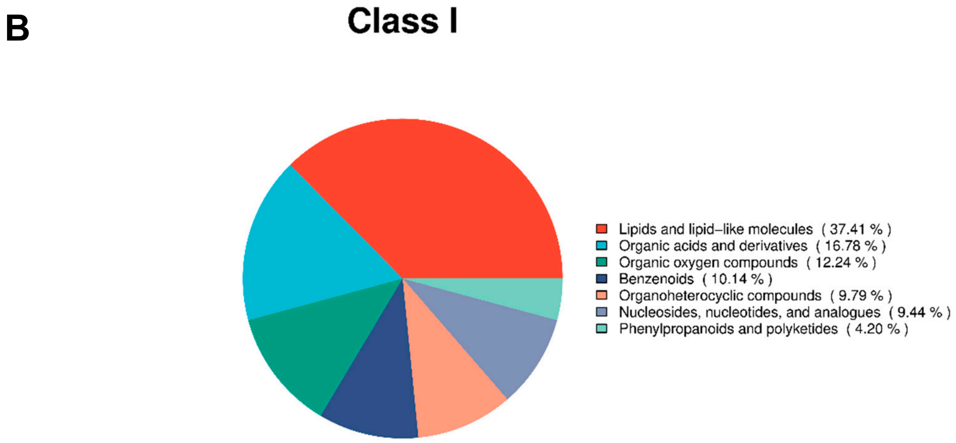

Figure S3. Pie chart of metabolite classification. In positive mode (A) and in negative mode (B).

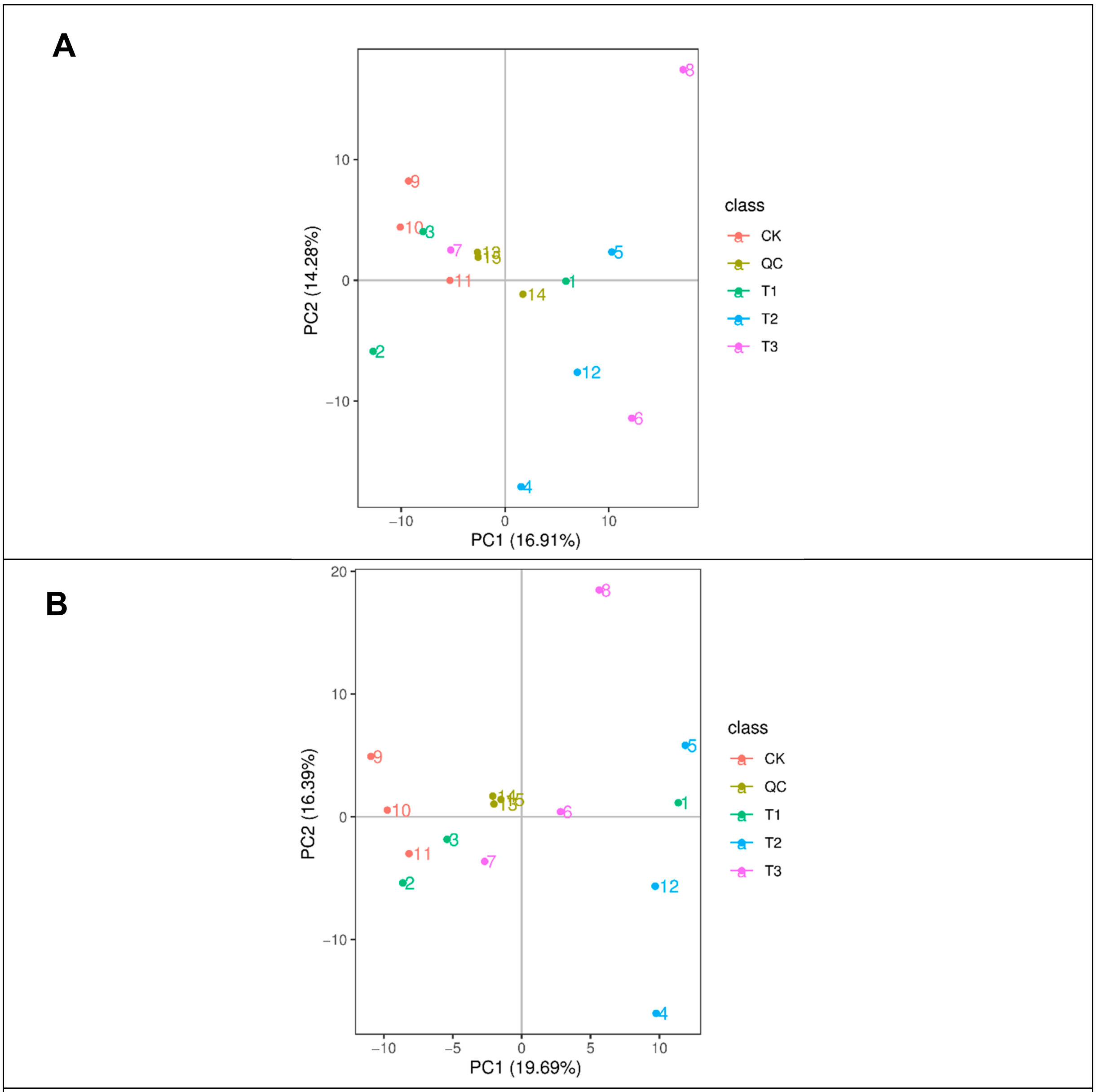

Figure S4. Principal component analysis was conducted on metabolites of different treatments and the metabolites that passed quality control. In positive mode (A) and in negative mode (B).

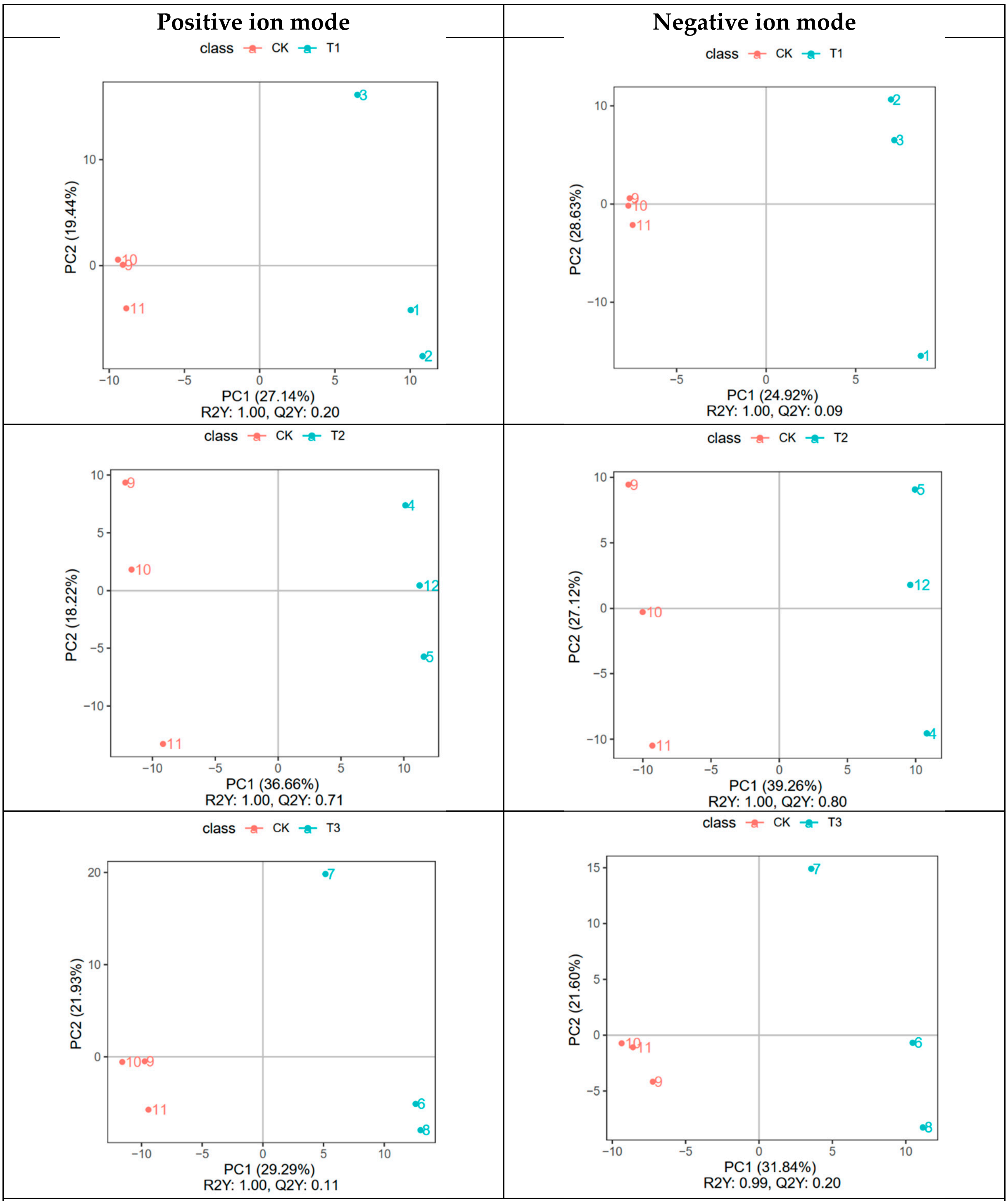

Figure S5. Partial least-squares discrimination analysis (PLS-DA) of different metabolites under different treatments.

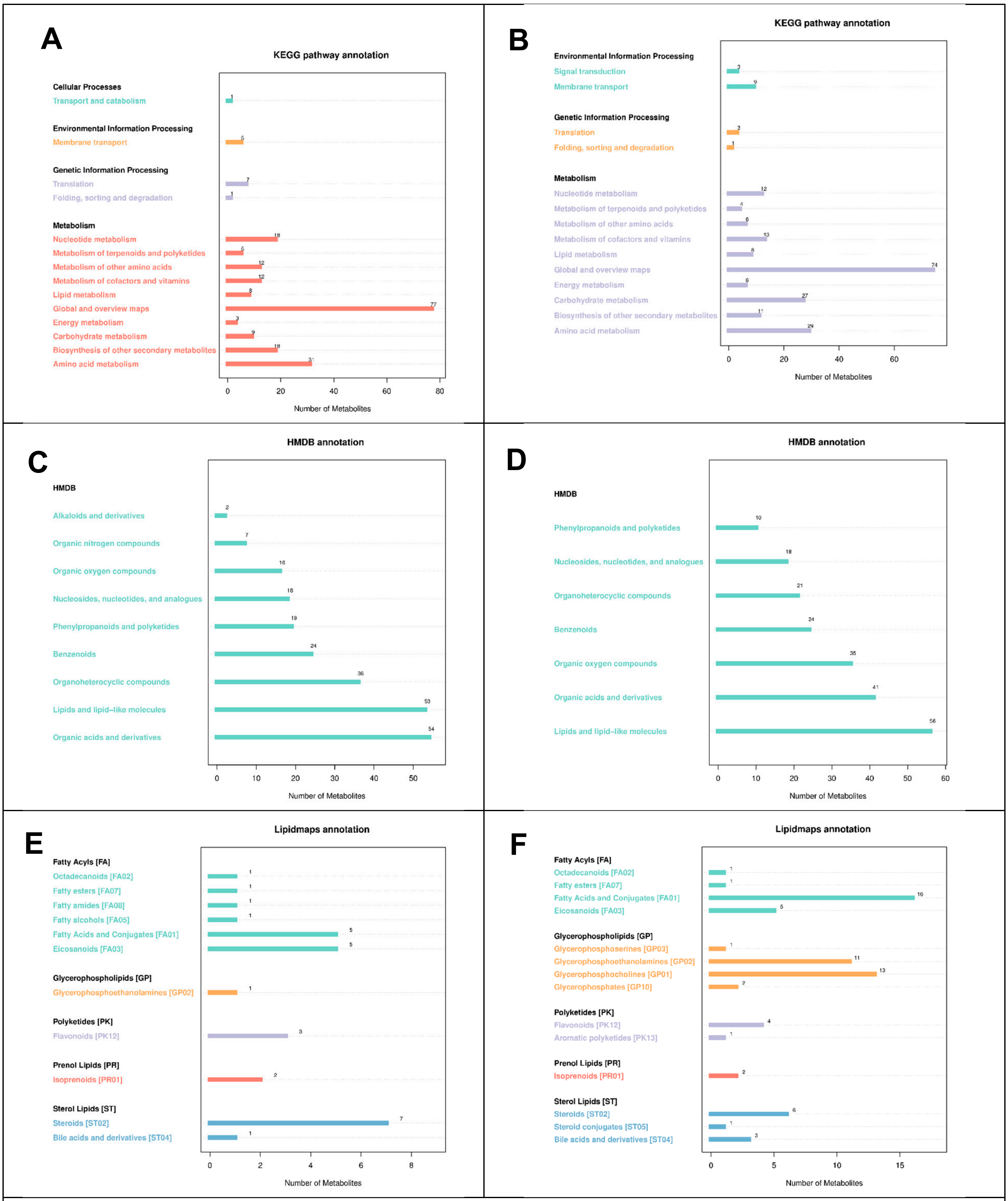

Figure S6. Function and classification of metabolites in three databases (KEGG、HMDB、LIPID MAPS). (A to C) In positive mode and (D to F) in negative mode.

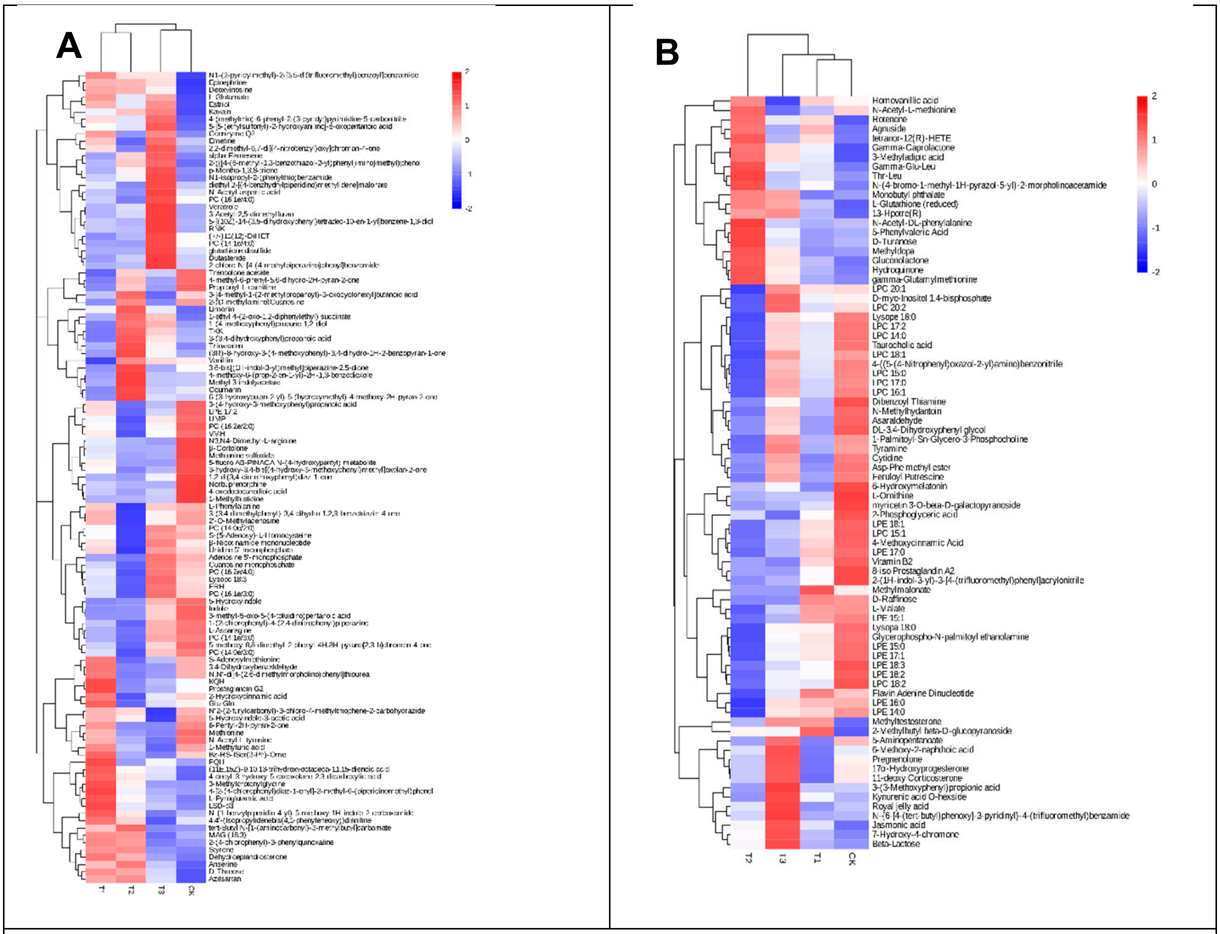

Figure S7. Hierarchical clustering analysis (HCA) of all differential expressed metabolites between different treatments. In positive mode (A) and in negative mode (B).

Table S1. Statistical table of 16S sequencing data.

Sample Name	Raw data	Effective tags	Q20	Q30	GC%	Effective%
CK.1	75,324	68,856	99.52	98.14	55.09	91.41
CK.2	74,274	63,251	99.46	97.93	55.17	85.16
CK.3	80,787	71,637	99.53	98.17	55.31	88.67
T1.1	75,567	69,001	99.63	98.44	56.41	91.31
T1.2	83,380	76,938	99.61	98.38	56.36	92.27
T1.3	80,853	72,993	99.66	98.55	56.52	90.28
T2.1	81,108	71,869	99.51	98.13	54.97	88.61
T2.2	79,269	69,285	99.57	98.28	55.53	87.4
T2.3	80,447	71,462	99.55	98.23	55.62	88.83
T3.1	59,910	55,592	99.54	98.23	52.91	92.79
T3.2	64,662	59,126	99.51	98.13	54.82	91.44
T3.3	79,496	70,742	99.52	98.16	54.65	88.99

Note: Raw date represents raw sequenced reads; Effective tags represent the reads used for subsequent analysis after quality control and filtration of chimeras. Q20 and Q30 are the percentage of bases with base mass values greater than 20 and 30 in the Effective tags; GC (%) indicates the content of GC bases in Effective tags; Effective (%) represents the percentage of the number of Effective tags versus the number of Raw data.

Table S2. Statistical table of ITS sequencing data.

Sample Name	Raw data	Effective tags	Q20	Q30	GC%	Effective%
CK.1	86,038	81,520	95.15	89.25	52.11	94.75
CK.2	84,207	79,829	96.18	90.82	51.87	94.8
CK.3	87,495	82,383	96.62	91.81	50.97	94.16
T1.1	85,537	79,627	96.12	91.16	51.24	93.09
T1.2	86,386	79,981	95.54	90.13	52.48	92.59
T1.3	80,749	77,326	97.17	93.3	51.46	95.76
T2.1	87,885	81,410	96.9	92.39	49.94	92.63
T2.2	84,323	79,135	96.54	91.53	50.42	93.85
T2.3	76,761	73,819	97.55	93.59	49.94	96.17
T3.1	86,399	83,126	98.71	96.39	46.98	96.21
T3.2	78,624	75,305	96.08	91.25	49.95	95.78
T3.3	78,249	75,985	98.82	96.52	46.34	97.11

Note: Raw date represents raw sequenced reads; Effective tags represent the reads used for subsequent analysis after quality control and filtration of chimeras. Q20 and Q30 are the percentage of bases with base mass values greater than 20 and 30 in the Effective tags; GC (%) indicates the content of GC bases in Effective tags; Effective (%) represents the percentage of the number of Effective tags versus the number of Raw data.

Table S3. The top 10 taxonomy relative abundance in bacterial phyla.

Taxonomy	T1	T2	T3	CK
Proteobacteria	0.323896c	0.447623b	0.53032a	0.446299b
Bacteroidota	0.061712c	0.104626b	0.18316a	0.129227b
Acidobacteriota	0.201628a	0.137425b	0.066514c	0.115477b
Gemmatimonadota	0.086648a	0.054488b	0.035097c	0.057458b
Cyanobacteria	0.026202b	0.023742b	0.033218a	0.030753a
Actinobacteriota	0.058562a	0.051732a	0.029248c	0.038705b
Chloroflexi	0.048356a	0.028545b	0.017255c	0.028209b
Firmicutes	0.008644b	0.021806a	0.004719b	0.026905a
Verrucomicrobiota	0.039267a	0.034064a	0.028099b	0.02981b
Planctomycetota	0.03573a	0.020863b	0.011232c	0.018539b
Others	0.109357a	0.075087b	0.061137b	0.078618b

Different lowercase letters within column show significant differences among treatments according to Duncan's multiple range test at $P < 0.05$.

Table S4. The top10 taxonomy relative abundance in fungal phyla.

Taxonomy	T1	T2	T3	CK
Ascomycota	0.268922c	0.363961b	0.447379a	0.262105c
Basidiomycota	0.026338c	0.042833b	0.153822a	0.046498b
Mortierellomycota	0.01637c	0.063697a	0.074859a	0.036645b
Chytridiomycota	0.001199b	0.005249a	0.00016c	0.002822b
Rozellomycota	0.00026c	0.003291a	0.001084b	0.0001c
Mucoromycota	0.001423b	0.001828a	0.00007c	0
Glomeromycota	0.001104b	0.001688a	0.00018c	0.000624c
Aphelidiomycota	0.00026b	0.000325a	0.00004c	0.000195b
Kickxellomycota	0.000025	0	0	0
Zoopagomycota	0	0.00002	0	0
Others	0.6841a	0.517109b	0.322407c	0.651011a

Different lowercase letters within column show significant differences among treatments according to Duncan's multiple range test at $P < 0.05$.

Table S5. Metabolite differential screening results.

Compared Samples	Total number	Number of Sig up	Number of Sig down
T1 vs CK-POS	26	13	13
T2 vs CK-POS	55	22	33
T3 vs CK-POS	29	18	11
T1 vs CK-NEG	13	2	11
T2 vs CK-NEG	50	14	36
T3 vs CK-NEG	16	10	6

POS represents in positive ion mode; NEG represents in negative ion mode; Sig up represents up-regulation; Sig down represents down-regulation.
